# The association between data-driven training monitoring and athlete burnout: a chain-mediated indirect path between performance anxiety and psychological exhaustion

**DOI:** 10.3389/fpsyg.2026.1893721

**Published:** 2026-10-02

**Authors:** Haoxuan Du, Pengzhou Chen

**Affiliations:** 1 Department of Physical Education, Fujian Business University, Fuzhou, China; 2 China Basketball College, Beijing Sport University, Beijing, China

**Keywords:** athlete burnout, data-driven training monitoring stress, depletion of psychological resources, performance anxiety, psychological exhaustion

## Abstract

**Introduction:**

Data-driven training monitoring has enhanced the precision of training-load assessment, fatigue detection, and training regulation in competitive sport. However, continuous data collection, performance metric comparisons, and evaluative feedback may also constitute emerging sources of psychological stress for athletes. Drawing on the Technology Stress Generators Model, the Challenge–Hindrance Stressor Framework, and the Job Demands–Resources Model, this study examined the relationships among data-driven training monitoring stress (DTMS), evaluative anxiety, psychological exhaustion, and athlete burnout, as well as potential differences across gender and sport type.

**Methods:**

Cross-sectional questionnaire data were collected from 588 active athletes. Confirmatory factor analysis, structural equation modeling, bootstrap tests of indirect effects, and multigroup analysis were conducted to examine the hypothesized relationships and between-group differences.

**Results:**

DTMS was significantly and positively associated with evaluative anxiety, psychological exhaustion, and athlete burnout. Evaluative anxiety and psychological exhaustion each constituted a significant indirect pathway between DTMS and athlete burnout, and the sequential indirect pathway through evaluative anxiety and psychological exhaustion was also statistically significant. Multigroup analysis further showed significant between-group differences in the association between DTMS and evaluative anxiety. This association was stronger among female athletes than among male athletes and stronger among athletes in physical endurance sports than among those in skill-based sports.

**Discussion:**

The findings suggest that data-driven training monitoring should extend beyond the technical objectives of data acquisition and assessment accuracy to incorporate psychologically informed practices. Particular attention should be given to the interpretation of monitoring data, the communication of anomalous indicators, and the delivery of evaluative feedback to reduce the psychological burden potentially associated with data-intensive monitoring.

## Introduction

As competitive sport training management increasingly shifts from experience-based judgment and periodic assessment toward real-time data acquisition, dynamic feedback, and data-informed decision-making, monitoring technologies have become deeply embedded in athletes’ daily training environments. Physiological and performance indicators, including heart rate, blood lactate concentration, and GPS-derived running metrics, are now widely used to assess training load, adjust training programs, and evaluate athletic performance (Bourdon et al., 2017; Saw et al., 2016; Dawson et al., 2024; Leduc and Weaving, 2025). From a performance-management perspective, data-driven monitoring has improved the precision of training-load assessment, fatigue detection, and injury-risk management, thereby facilitating a shift from generalized training prescriptions toward more individualized regulation. Nevertheless, the increasing integration of data collection, metric comparison, and evaluative feedback into athletes’ performance, recovery, and training behaviors may also introduce new psychological demands. Athletes are required not only to complete prescribed training tasks but also to adapt continuously to having their physical condition recorded, performance quantified, recovery status tracked, and individual differences compared. Accordingly, data-driven training monitoring stress may provide an important conceptual lens through which to understand athletes’ psychological adaptation and vulnerability to burnout.

Existing research has consistently emphasized the contribution of training monitoring to performance enhancement and training-load management, particularly through the integration of multiple data sources to improve the accuracy and timeliness of training decisions (Soligard et al., 2016; Bourdon et al., 2017; Dawson et al., 2024). In practice, however, data-driven monitoring is not a psychologically neutral technical arrangement. Its psychological implications depend on how monitoring is implemented, how results are communicated, how data are incorporated into evaluation, and how athletes interpret the monitoring process. When monitoring outcomes are repeatedly linked to ranking, team selection, punitive consequences, increased training demands, or qualification decisions, the resulting data may acquire evaluative significance and become salient signals of judgment. Under such conditions, monitoring may be associated with heightened evaluative anxiety, fear of unfavorable outcomes, and defensive psychological responses. Research on technostress has shown that continuous information tracking, increased performance visibility, and persistent demands for technological adaptation can contribute to cognitive overload and psychological strain (Tarafdar et al., 2007). More recently, a cross-sectional study in a sport context reported significant associations between technology-related stress, cognitive fatigue, and athlete burnout, further highlighting the potential psychological burden associated with digital technology use in training environments (Pan et al., 2026). Research on athlete burnout likewise indicates that persistently demanding training environments characterized by high stress, inadequate recovery, and elevated performance expectations are closely associated with emotional and physical exhaustion, reduced athletic accomplishment, and sport devaluation (Raedeke, 1997; Gustafsson et al., 2017). Recent evidence also suggests that the association between burnout and athletic performance warrants closer examination (Olsson et al., 2025).

Taken together, these findings indicate that the significance of data-driven training monitoring extends beyond improvements in managerial efficiency and decision accuracy. It also concerns the ways in which athletes’ psychological resources are mobilized, taxed, and potentially depleted within increasingly data-intensive training environments. Although emerging research has begun to examine technology-related stress and its associations with cognitive fatigue and athlete burnout (Pan et al., 2026), most studies on training monitoring continue to focus primarily on load assessment, injury prevention, performance optimization, and feedback effectiveness. Comparatively less attention has been devoted to the psychological pressure generated by continuous data collection and, more importantly, by the evaluative use of monitoring information. This omission is noteworthy for two reasons. First, training monitoring is commonly framed as an indispensable component of scientific training, meaning that its potential psychological costs may be obscured by dominant narratives of precision, objectivity, and technological empowerment. In high-performance training systems, athletes may continue to comply with intensive monitoring despite experiencing anxiety or resistance because training participation, competition opportunities, and team status may depend on such compliance. Second, existing research has tended to examine either general forms of technostress or athlete burnout as relatively broad constructs, providing limited insight into how training data may become psychologically consequential when embedded in evaluative processes such as ranking, selection, coaching judgments, and opportunity allocation. The specific pathway linking monitoring-related pressure to evaluative anxiety, psychological resource depletion, and burnout therefore remains insufficiently understood.

To address these theoretical and practical gaps, the present study focuses on athletes operating within competitive training environments and conceptualizes data-driven training monitoring stress as a distinct psychological demand arising from digitally mediated training management. Evaluative anxiety and psychological exhaustion are introduced as two key psychological mechanisms through which monitoring-related stress may be associated with athlete burnout. On this basis, the study develops and tests a theoretical pathway linking data-driven training monitoring stress, evaluative anxiety, psychological exhaustion, and athlete burnout. Using cross-sectional questionnaire data and structural equation modeling, the study examines whether continuous data collection, metric comparison, and evaluative feedback are associated with a pattern of relationships consistent with theoretical expectations regarding negative evaluation, psychological resource depletion, and burnout. In doing so, the study seeks to clarify the psychological processes through which data-intensive monitoring may become consequential for athletes and to extend existing explanations of athlete burnout beyond conventional emphases on training load, insufficient recovery, and motivational quality.

## Theoretical foundations and research hypotheses

### Theoretical foundations

#### Technological generation of data-driven training monitoring stress: the technostress creators model

The Technology Stress Generators Model provides an important theoretical framework for explaining the psychological consequences that arise when information technologies become deeply embedded in organizational processes. The model proposes that the introduction of technological systems can reshape how individuals process information, organize tasks, respond to feedback, and manage role expectations. Technostress is likely to emerge when technological demands exceed individuals’ adaptive capacity or when technology persistently increases the cognitive and psychological burden associated with information processing, performance monitoring, and feedback management. Tarafdar et al. (2007) identified five major technostress creators–techno-overload, techno-invasion, techno-complexity, techno-insecurity, and techno-uncertainty–highlighting how persistent technological demands can intensify psychological strain. Extending this perspective, Ragu-Nathan et al. (2008) further emphasized that technostress reflects a misalignment between technological demands, organizational expectations, and individuals’ capacity to adapt. Taken together, this framework explains how technologies embedded in organizational settings may influence individuals’ psychological functioning through intensified demands, increased performance visibility, and continuous requirements for adaptation.

When applied to competitive sport, data-driven training monitoring provides a comparable context in which technology-related stress may emerge. Monitoring systems transform athletes’ physiological states, training responses, and recovery processes into continuously traceable indicators through measures such as heart rate, blood lactate concentration, GPS-derived workload, and other performance metrics. Although these data are primarily intended to support training adjustment and performance management, their continuous integration into daily training routines, coach feedback, and team evaluation processes can substantially increase the visibility of athletes’ performance. As a result, athletes train in an environment in which their physical condition is continuously recorded, their performance outcomes are repeatedly communicated, and individual differences are increasingly rendered comparable through quantitative indicators.

The psychological pressure associated with data-driven training monitoring arises particularly from the continuous involvement of technological systems in the evaluation and regulation of training. Continuous data acquisition makes fluctuations in athletes’ physical condition more readily identifiable, converting states that were previously contextual, transient, or difficult to observe into explicit and traceable information. Metric-based feedback further increases the evaluative salience of training outcomes. When monitoring data are incorporated into coaching judgments, training adjustments, competition selection, or team rankings, such data may acquire consequences that extend beyond technical assessment and become salient signals of evaluation. At the same time, monitoring systems increase the adaptive demands placed on athletes. Athletes must interpret the meaning of performance indicators, respond to changes in metrics, make sense of anomalous results, and adjust their training behavior in accordance with ongoing feedback. As the cycle of data acquisition, evaluative feedback, and behavioral adjustment becomes increasingly continuous, monitoring technology may shift from functioning primarily as an external support tool to becoming a persistent source of psychological demand embedded within the training system.

Data-driven training monitoring stress is theoretically related to several established constructs, including general technostress, performance pressure, evaluation pressure, coach supervision, and fear of negative evaluation. Nevertheless, its conceptual focus and conditions of emergence are not identical to those of these constructs. General technostress primarily concerns psychological strain arising from technological overload, complexity, intrusion, insecurity, and adaptation demands across a wide range of technological environments rather than specifically within competitive training. Performance pressure and evaluation pressure emphasize demands associated with achievement expectations and external judgment, but they do not necessarily require continuous digital data collection, quantitative representation, or real-time performance visibility. Coach supervision primarily concerns external observation, oversight, and behavioral control by coaches or training managers, whereas fear of negative evaluation reflects individuals’ cognitive and affective sensitivity to the possibility of unfavorable judgment by others.

In contrast, data-driven training monitoring stress refers specifically to the situational psychological pressure that arises when athletes’ physical condition and training performance are continuously recorded, quantified, compared, and incorporated into training feedback and evaluative processes. Its distinctive feature lies in the technological transformation of training performance from a context-dependent and temporally bounded experience into a continuously visible, traceable, and comparable stream of data. This transformation increases the demands placed on athletes to interpret monitoring information, respond to anomalous indicators, adapt to repeated feedback, and manage the evaluative consequences associated with quantified performance. Accordingly, the present study does not conceptualize data-driven training monitoring stress as a wholly novel construct that is entirely independent of existing stress-related concepts. Rather, it defines DTMS as a context-specific form of psychological demand characterized by a distinctive technological basis, evaluative mode, and set of adaptation requirements within digitally mediated competitive training environments.

#### Hindrance transformation of data-driven evaluation stress: the Challenge–Hindrance Stressor Framework

The Challenge–Hindrance Stressor Framework provides an important theoretical perspective for understanding heterogeneity in individuals’ responses to stressors. Cavanaugh et al. (2000) proposed that stressors do not exert uniform effects on psychological and behavioral outcomes; rather, their consequences depend partly on how individuals appraise the relationship between situational demands and goal attainment. Stressors perceived as facilitating competence development, goal achievement, and personal growth are generally conceptualized as challenge stressors, whereas demands perceived as constraining development, reducing control, or obstructing goal attainment are classified as hindrance stressors. Extending this distinction, LePine et al. (2005) showed that challenge stressors, although potentially associated with strain, may also be related to favorable behavioral outcomes by stimulating effort and motivation. Hindrance stressors, by contrast, are more consistently associated with psychological strain, negative affect, and impaired performance. This framework therefore moves beyond the assumption that all stressors are inherently detrimental and instead emphasizes the importance of individuals’ appraisals of the meaning and implications of specific demands.

Within the context of data-driven training monitoring, monitoring data do not inherently constitute a hindrance stressor. Appropriate feedback can help athletes assess their physical condition, understand training responses, refine technical execution, and optimize recovery strategies, thereby allowing monitoring demands to acquire challenge-related characteristics. When monitoring information is interpretable, actionable, and oriented toward training improvement, athletes may perceive it as a useful resource for enhancing performance and facilitating adaptation. However, when monitoring results become closely linked to repeated rankings, competition selection decisions, additional training demands, or coaching evaluations, their meaning may shift from developmental support toward evaluative constraint. Under such conditions, athletes are confronted not merely with objective information about their physical condition but with quantified signals that carry potential evaluative consequences. Deviations, deteriorations, or anomalous indicators may therefore be interpreted as evidence of insufficient ability, inadequate effort, or poor adaptation to training.

This shift in appraisal represents an important mechanism through which data-driven monitoring may generate evaluative pressure. By increasing the visibility and comparability of the training process, monitoring systems make fluctuations in athletes’ physical condition easier to detect and evaluate. At the same time, short-term errors, fatigue responses, or temporary limitations in recovery may become more difficult to interpret within their broader situational context once they are represented as explicit quantitative indicators. When monitoring feedback is incorporated into training management in a frequent, highly comparative, and outcome-oriented manner, athletes may become increasingly concerned about whether their indicators meet expected standards, whether anomalous results will affect coaching judgments, and whether monitoring outcomes will lead to additional training demands or disadvantages in competition selection. Under these conditions, monitoring-related demands may increasingly be appraised in terms of their evaluative consequences. Evaluative anxiety may consequently emerge as anticipatory concern regarding unfavorable judgments, loss of status, or the loss of valued opportunities.

The Challenge–Hindrance Stressor Framework therefore provides a useful theoretical basis for explaining the potentially divergent psychological consequences of data-driven training monitoring. Although monitoring can contribute positively to training adjustment, performance development, and risk detection, its psychological implications are not determined solely by technical accuracy. They also depend on how monitoring information is interpreted, how feedback is communicated, and how data are incorporated into broader systems of evaluation and decision-making. When feedback identifies opportunities for improvement and supports competence development, monitoring-related demands may be appraised as challenges. In contrast, when monitoring results are persistently used for comparison, selection, punitive consequences, or the attribution of responsibility, the same demands are more likely to acquire hindrance characteristics. From this perspective, the evaluative use of monitoring data provides a theoretical basis for explaining why data-driven training monitoring stress may be associated with evaluative anxiety and, subsequently, with psychological exhaustion and athlete burnout.

#### Demand–resource imbalance in athlete burnout: the Job Demands–Resources Model

The Job Demands–Resources (JD–R) Model, originally proposed by Demerouti et al. (2001) and subsequently developed by Bakker and Demerouti (2007), provides an influential theoretical framework for explaining the associations among sustained demands, exhaustion, and burnout. The model distinguishes between job demands, which require sustained physical or psychological effort, and job resources, which facilitate goal attainment, reduce the costs associated with demands, and support personal development. When demands remain persistently high while available psychological, social, or organizational resources are insufficient to sustain effective adaptation, individuals may experience progressive energy depletion and, ultimately, adverse outcomes such as exhaustion, disengagement, and diminished efficacy. Accordingly, the JD–R Model conceptualizes burnout as an outcome associated with a sustained imbalance between excessive demands and insufficient resources.

Applied to competitive sport, the JD–R perspective suggests that athletes operate within demanding environments characterized by the simultaneous presence of intensive training requirements and supportive resources. High training loads, frequent competition, and persistent performance expectations constitute major demands that athletes must manage, whereas coaching support, adequate recovery opportunities, and psychological safety represent important resources that facilitate adaptation. The increasing integration of data-driven monitoring may add a further layer of psychological demand to this environment. In addition to completing prescribed training tasks, athletes may be required to respond continuously to monitoring feedback, interpret fluctuations in performance indicators, manage comparisons with teammates or normative standards, and maintain stable performance when monitoring outcomes are incorporated into evaluation and opportunity allocation.

Evaluative anxiety may represent an important psychological response when monitoring-related demands become salient and available resources are insufficient to offset them. When athletes perceive monitoring data as an important basis for coaching judgments, feedback may become associated with anticipatory concerns about unfavorable evaluation. Athletes may repeatedly assess whether their indicators meet expected standards, worry that anomalous results will be interpreted as evidence of inadequate effort or declining ability, and regulate their observable behavior in an effort to maintain a favorable impression. Such persistent evaluative concerns may consume attentional and self-regulatory resources, potentially reducing the resources available for task execution, technical refinement, and awareness of bodily cues during training. When these demands recur over time, opportunities for psychological recovery and adaptive engagement may become increasingly constrained, creating conditions under which psychological exhaustion may accumulate.

Within the JD–R framework, psychological exhaustion represents a key state associated with sustained resource depletion under conditions of chronic demand. The model emphasizes that the consequences of excessive demands generally emerge through a gradual process of energy depletion rather than as an immediate response. In data-intensive training environments, repeated monitoring, performance comparisons, and evaluative feedback may require sustained vigilance, interpretation, and self-regulation, thereby placing persistent demands on athletes’ psychological resources. Psychological exhaustion therefore extends beyond transient fatigue and reflects a more persistent experience of depleted psychological capacity following prolonged exposure to demanding conditions. It may manifest as mental fatigue before training, difficulty sustaining attention during training, impaired psychological recovery after training, and reduced readiness to engage in subsequent tasks. Even when athletes remain behaviorally compliant with training requirements, their emotional vitality and motivational resources may already be substantially diminished.

The JD–R Model thus provides a systematic theoretical basis for explaining why data-driven training monitoring stress may be associated with athlete burnout. Although monitoring technologies can improve the precision and responsiveness of training management, they may simultaneously increase psychological demands related to evaluation, feedback processing, and self-regulation. When these demands are not adequately balanced by interpretable feedback, emotional support, recovery opportunities, and other relevant resources, athletes may be more likely to exhibit a pattern characterized by heightened evaluative anxiety, greater psychological exhaustion, and more pronounced burnout symptoms. From this perspective, data-driven training monitoring stress can be conceptualized as a context-specific psychological demand within digitally mediated training environments, while psychological exhaustion represents an important resource-depletion mechanism through which its association with athlete burnout can be theoretically understood.

### Research hypotheses

Based on the theoretical foundations outlined above, the present study conceptualizes data-driven training monitoring stress (DTMS) as a context-specific psychological demand arising from increasingly data-intensive training environments. Evaluative anxiety and psychological exhaustion are proposed as two key psychological mechanisms linking DTMS to athlete burnout. Accordingly, the study develops a theoretical pathway model in which DTMS is associated with evaluative anxiety, psychological exhaustion, and athlete burnout, while evaluative anxiety and psychological exhaustion further constitute indirect pathways linking DTMS to burnout.

Data-driven training monitoring stress refers to the situational psychological pressure experienced by athletes when continuous data collection, metric comparison, performance feedback, and the evaluative use of monitoring information become embedded in daily training. When monitoring outcomes are linked to rankings, team selection, training adjustments, competition opportunities, or coaching evaluations, athletes may become increasingly concerned about unfavorable judgments and the potential consequences of anomalous or suboptimal data. Persistent evaluative concerns may, in turn, place continuing demands on attentional and self-regulatory resources, thereby contributing to psychological exhaustion and more pronounced burnout symptoms. Given potential differences in evaluative experiences, performance demands, monitoring intensity, and sport-specific uses of training data, the study further examines whether key structural relationships vary by gender and sport type.

**H1. Direct Associations of Data-Driven Training Monitoring Stress**

The Technology Stress Generators Model suggests that psychological strain may arise when technological systems continuously alter task demands, information-processing requirements, feedback processes, and evaluative environments. In competitive training, data-driven monitoring increases the frequency with which athletes’ physiological condition, training performance, and recovery status are recorded, quantified, and evaluated. Athletes must therefore manage not only prescribed training tasks but also ongoing data fluctuations, anomalous indicators, comparative feedback, and the potential evaluative consequences associated with monitoring outcomes. Such demands may be associated with heightened evaluative concerns, greater psychological vigilance, increased self-monitoring, and greater depletion of psychological resources.

H1a: Higher levels of DTMS are associated with higher levels of evaluative anxiety.

H1b: Higher levels of DTMS are associated with higher levels of psychological exhaustion.

H1c: Higher levels of DTMS are associated with higher levels of athlete burnout.

**H2. Associations of Evaluative Anxiety With Psychological Exhaustion and Athlete Burnout**

The Challenge–Hindrance Stressor Framework emphasizes that the psychological consequences of stressors depend partly on how individuals appraise their relevance to goal attainment and their potential consequences. Data-driven monitoring can function as a challenge-related demand when it supports training improvement, workload adjustment, and injury-risk detection. However, when monitoring results are closely tied to coaching evaluations, intra-team comparisons, increased training demands, or competition opportunities, athletes may increasingly interpret data feedback as carrying unfavorable evaluative consequences.

Under such conditions, athletes may become preoccupied with whether their indicators meet expected standards and whether anomalous results will be interpreted as evidence of inadequate ability, poor condition, or insufficient commitment to training. Evaluative anxiety therefore represents an important psychological response to monitoring-related demands. Persistent concern about negative evaluation may require continued emotional regulation, impression management, and behavioral adjustment, thereby placing additional demands on attentional and self-regulatory resources. Consequently, higher levels of evaluative anxiety may be associated with greater psychological exhaustion and more pronounced burnout symptoms.

H2a: Higher levels of evaluative anxiety are associated with higher levels of psychological exhaustion.

H2b: Higher levels of evaluative anxiety are associated with higher levels of athlete burnout.

**H3. Association Between Psychological Exhaustion and Athlete Burnout**

The Job Demands–Resources Model proposes that sustained exposure to high demands in the absence of sufficient compensatory resources is associated with progressive energy depletion and burnout. Competitive training is inherently characterized by intensive workloads, persistent performance expectations, frequent evaluation, and competitive pressure. The integration of data-driven monitoring may add further demands by requiring athletes to attend continuously to performance indicators, interpret data fluctuations, compare outcomes, and respond to anomalous results.

When such monitoring and evaluative demands persist, repeated vigilance, self-regulation, and behavioral adjustment may place continuing pressure on athletes’ psychological resources. Psychological exhaustion reflects this depleted state and may be accompanied by reduced concentration, lower emotional vitality, impaired recovery, diminished perceptions of accomplishment, and increasing detachment from sport. Accordingly, greater psychological exhaustion is expected to be associated with greater athlete burnout.

H3a: Higher levels of psychological exhaustion are associated with higher levels of athlete burnout.

H4. Indirect Associations Through Evaluative Anxiety and Psychological Exhaustion

The association between DTMS and athlete burnout may extend beyond direct relationships and involve indirect psychological pathways. Evaluative anxiety reflects athletes’ anticipatory concerns that monitoring outcomes may result in unfavorable judgments, diminished status, or lost opportunities. It therefore represents an evaluative response through which monitoring-related stress may be associated with burnout. Psychological exhaustion, in contrast, reflects a state of depleted psychological resources associated with sustained demands for vigilance, interpretation, self-regulation, and adaptation. It therefore provides a resource-depletion mechanism through which DTMS may also be indirectly associated with burnout.

H4a: DTMS has a significant indirect association with athlete burnout through evaluative anxiety.

H4b: DTMS has a significant indirect association with athlete burnout through psychological exhaustion.

**H5. Sequential Indirect Association Through Evaluative Anxiety and Psychological Exhaustion**

The preceding arguments further suggest a sequential psychological pathway. Higher levels of DTMS may be associated with stronger expectations of unfavorable evaluation and, consequently, greater evaluative anxiety. Persistent evaluative anxiety may require athletes to devote additional attentional and self-regulatory resources to interpreting monitoring information, controlling emotional responses, adjusting training behavior, and maintaining a favorable performance image. These continuing demands may be associated with greater psychological exhaustion. In turn, higher levels of psychological exhaustion may coincide with diminished positive training experiences, reduced perceived efficacy, greater fatigue, and stronger sport-related detachment.

Accordingly, DTMS may be indirectly associated with athlete burnout through a sequential pathway involving evaluative anxiety and psychological exhaustion.

H5a: DTMS has a significant sequential indirect association with athlete burnout through evaluative anxiety and psychological exhaustion.

**H6. Group Differences by Gender and Sport Type**

The strength of the association between DTMS and evaluative anxiety may vary across athlete groups because monitoring practices and evaluative experiences are not uniform. Gender-related differences may reflect variation in experiences of performance evaluation, concerns about physical presentation, responsiveness to feedback, and sensitivity to continuous quantitative comparison. Because data-driven monitoring increases the visibility, comparability, and traceability of training performance, athletes of different genders may differ in the extent to which monitoring-related demands are associated with evaluative anxiety. The present study therefore examines gender differences in the DTMS–evaluative anxiety pathway without assuming a specific directional pattern.

Sport type may also shape the psychological meaning of data-driven monitoring because sports differ in the extent to which quantitative indicators are embedded in training decisions and performance evaluation. In this study, sports are operationally categorized as physical endurance sports, skill-based sports, and competitive ball sports according to the relative importance of quantitative monitoring indicators, the extent to which monitoring data directly inform training decisions and performance evaluations, and the extent to which performance depends on technical, tactical, and situational factors. Physical endurance sports typically rely heavily on continuously quantified indicators such as heart rate, speed, distance, workload, and recovery status, and these measures may be directly incorporated into training adjustments and assessments of competitive condition. Skill-based sports also use monitoring technologies, but evaluation tends to depend more strongly on movement quality, technical difficulty, and execution consistency, with quantitative indicators often serving a supplementary role. Competitive ball sports integrate physical workload data with technical, tactical, positional, and situational information, resulting in a more complex relationship between monitoring data and performance evaluation. Accordingly, the evaluative significance of monitoring data may differ across sport types, potentially producing group differences in the strength of the DTMS–evaluative anxiety association.

H6a: The association between DTMS and evaluative anxiety differs by gender.

H6b: The association between DTMS and evaluative anxiety differs across sport types.

In summary, the hypothesized model tested in the present study is presented in Figure 1.

**FIGURE 1:**
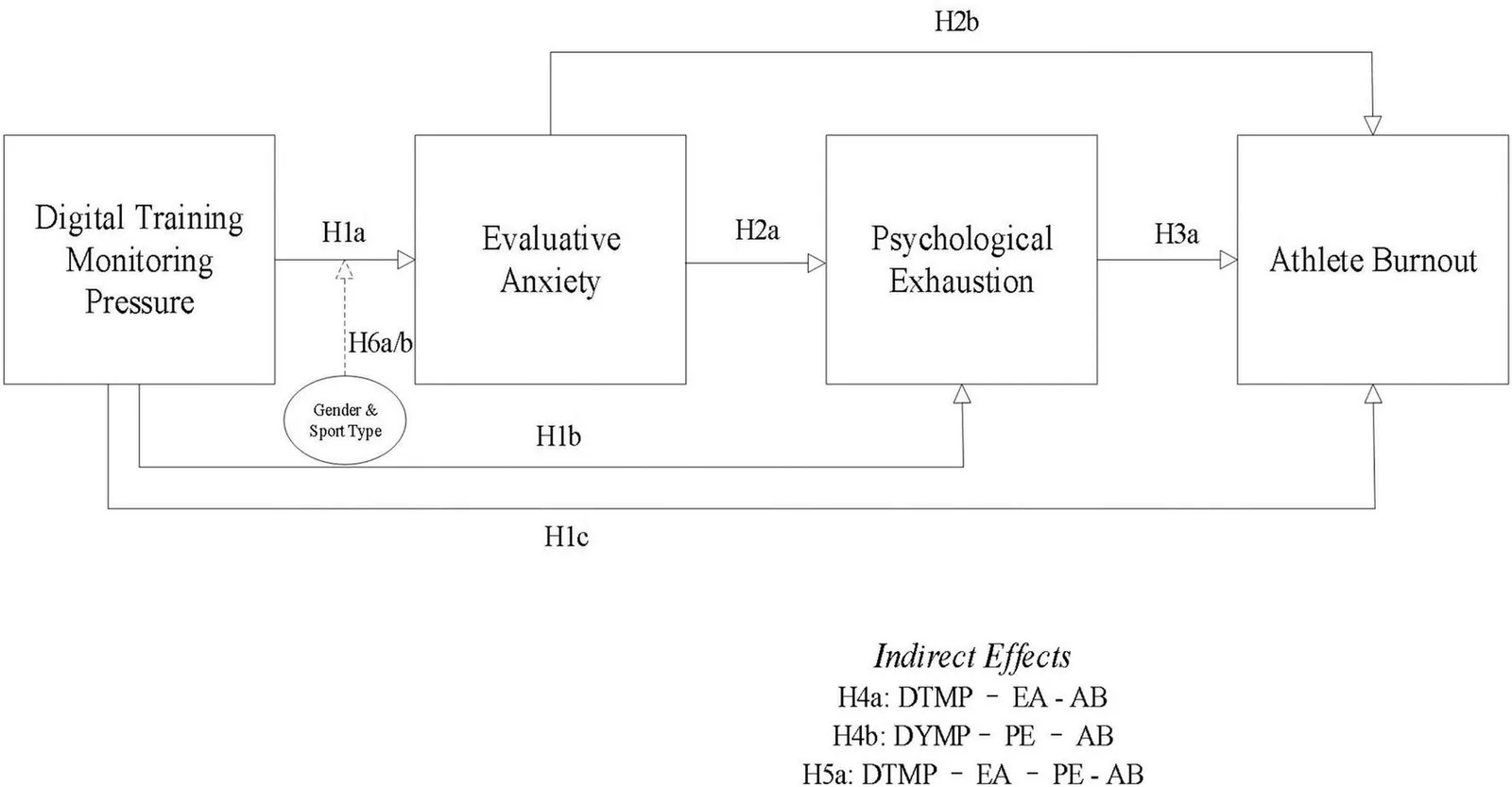
Conceptual model of the study. DTMP, digital training monitoring pressure; EA, evaluative anxiety; PE, psychological exhaustion; AB, athlete burnout.

## Research design

### Study population and sample sources

This study employed a cross-regional, multi-setting recruitment strategy and targeted active athletes within China’s competitive sport training system. Participants were primarily recruited from high-level university sports teams, provincial and municipal professional teams, and selected competitive sport reserve teams engaged in long-term systematic training. The sample encompassed athletes from different sport types, competitive levels, and stages of athletic development. The inclusion criteria were as follows: participants were required to be actively engaged in systematic sport-specific training for at least 1 year; to participate in sport-specific training at least four times per week; to belong to teams that routinely used data-driven monitoring methods, such as heart rate monitoring, training-load tracking, GPS-derived distance, recovery-status assessment, or subjective fatigue monitoring; and to be able to understand the questionnaire and voluntarily participate in the study. Athletes who had been absent from systematic training for an extended period because of injury, had recently discontinued systematic training, or provided substantially incomplete questionnaire responses were excluded from the final analysis. This population was selected because these athletes are routinely exposed to training environments characterized by high training loads, frequent evaluation, and intensive process monitoring, making them particularly relevant for examining the associations among data-driven training monitoring stress, evaluative anxiety, psychological exhaustion, and athlete burnout.

The survey covered five regions of China–East China, North China, Central China, Southwest China, and South China–and included nine provincial-level administrative regions: Jiangsu, Shandong, Tianjin, Hebei, Henan, Sichuan, Guangxi, Fujian, and Yunnan. The final sample comprised 260 athletes (44.2%) from high-level university sports teams, 190 athletes (32.3%) from provincial and municipal professional teams, and 138 athletes (23.5%) from competitive sport reserve teams. Recruitment was conducted primarily through participating teams and training organizations, with researchers or designated team coordinators inviting athletes who met the inclusion criteria to complete the survey. Because the study did not employ probability-based sampling from a national athlete registry, the sample was intended primarily for testing theoretical associations among the study variables rather than for estimating population prevalence among Chinese athletes. Restricting the sample to athletes within a relatively consistent national training context also reduced potential heterogeneity associated with cross-cultural differences in training systems and allowed the analysis to focus more directly on the relationships between monitoring-related stress and burnout within digitally mediated training environments.

Before the formal survey, the research team conducted a pilot test with 60 active athletes to assess item comprehensibility, contextual appropriateness, and questionnaire completion time. Based on pilot feedback, several items with overlapping meanings or ambiguous wording were revised. Administration instructions were also standardized, including statements regarding anonymity and the absence of correct or incorrect responses. The formal survey was administered through a combination of online questionnaires and in-person group administration. Researchers or trained team coordinators explained the purpose of the study, the principles of anonymity, and the response requirements before participants completed the questionnaire. No personally identifiable information, including names or identification numbers, was collected, and all data were used exclusively for academic research. Because the anonymized dataset retained only participants’ team categories and did not include hierarchical identifiers for specific teams or training organizations, clustering effects at the team level could not be estimated. Accordingly, the individual athlete was treated as the unit of statistical analysis.

A total of 680 questionnaires were distributed, of which 647 were returned, yielding a questionnaire return rate of 95.1%. Following data screening, 59 questionnaires were excluded because of excessive consecutive identical responses, substantial missing data, implausible completion times, or inconsistencies in logical checks. The final analytic sample therefore comprised 588 valid questionnaires, corresponding to a valid-response rate of 90.9% among returned questionnaires. The resulting sample size was adequate for the planned structural equation modeling and multigroup analyses and provided a sufficient basis for examining indirect associations involving evaluative anxiety and psychological exhaustion, as well as group differences across gender and sport type.

Before formal data collection, the study received approval from the Ethics Review Committee of Fujian University of Commerce (Approval No. FJBU-2026-26). All participants took part voluntarily after providing informed consent. For participants younger than 18 years, participation procedures followed the requirements specified in the approved ethics protocol, including the relevant consent or authorization procedures for minors. The study was conducted in accordance with the Declaration of Helsinki and applicable ethical guidelines. Participation was voluntary, questionnaire responses were anonymous, and data confidentiality was maintained throughout the research process. No personally identifiable information was included in the reporting of the results.

In the final sample, participants ranged in age from 16 to 25 years, with a mean age of 19.94 years (SD = 2.17). The sample included 316 male athletes (53.7%) and 272 female athletes (46.3%). By sport type, 198 athletes (33.7%) participated in physical endurance sports, 187 (31.8%) in skill-based sports, and 203 (34.5%) in competitive ball sports. In terms of athletic classification, 139 athletes (23.6%) held a National Class 1 ranking or higher, 261 (44.4%) held a National Class 2 ranking, and the remaining 188 (32.0%) were either unranked or had not yet received an official classification. Participants had engaged in sport-specific training for an average of 7.41 years (SD = 3.06). Weekly training frequency averaged 6.42 sessions (SD = 1.28), with most athletes training between five and eight times per week. Most participating teams routinely used one or more forms of digital training monitoring. Common practices included heart rate monitoring, training-load tracking, subjective fatigue assessment, running distance or speed monitoring, and recovery-status evaluation, indicating that the sample was well aligned with the data-driven training monitoring context examined in the present study.

### Measurement instruments

This study employed a structured questionnaire to assess data-driven training monitoring stress (DTMS), evaluative anxiety, psychological exhaustion, and athlete burnout. All items measuring the core constructs were rated on a 5-point Likert scale ranging from 1 (strongly disagree) to 5 (strongly agree). Scores for each construct were calculated by averaging the corresponding item scores, with higher scores indicating higher levels of the respective construct.

For instruments developed or contextually adapted for competitive training settings, the study followed a sequential procedure comprising theoretical construct specification, item generation and contextual adaptation, expert review, pilot testing, and psychometric evaluation using the formal sample. First, the conceptual domain of each construct was defined on the basis of relevant theoretical frameworks and previous research, after which candidate items were generated or adapted accordingly. Three experts in sport training and sport psychology subsequently evaluated the items in terms of content relevance, clarity, and contextual appropriateness. Based on their feedback, items with an ambiguous conceptual focus, semantic redundancy, or insufficient contextual relevance were revised. A pilot test involving 60 active athletes was then conducted to assess item comprehensibility, questionnaire completion time, preliminary item discrimination, and reliability. Items exhibiting ambiguous wording, substantial semantic overlap, or inadequate discrimination were revised or removed. Following formal data collection, the psychometric properties of the retained items were further examined through item analysis, confirmatory factor analysis, and assessments of reliability, convergent validity, and discriminant validity. At the conceptual level, particular attention was given to distinguishing situational monitoring-related demands, evaluative psychological responses, psychological resource depletion, and athlete burnout to minimize construct overlap among theoretically adjacent variables.

Data-driven training monitoring stress was assessed as athletes’ subjective psychological pressure arising from continuous data collection, metric comparison, evaluative feedback, and the use of monitoring outcomes in training management. Because existing measures do not adequately capture the specific demands associated with data-driven monitoring in competitive training, the study did not directly adopt a general technostress scale. Instead, a context-specific measure was developed with reference to the Technology Stress Generators Model, previous research on training monitoring, and insights derived from athlete interviews (Tarafdar et al., 2007; Ragu-Nathan et al., 2008; Bourdon et al., 2017).

The item-development framework focused on three theoretically relevant domains: continuous monitoring exposure arising from repeated data recording; evaluative demands associated with metric comparison and outcome feedback; and adaptive demands arising from the interpretation of and response to anomalous data. These domains correspond to three defining characteristics of DTMS: continuous visibility, evaluative demands, and requirements for ongoing adaptation. Following expert review and pilot-based revision, eight items were retained. Example items included: “The continuous recording of my training data causes me stress,” “When my training data fall short of expectations, I worry that my coach will judge my condition negatively,” and “I need to expend additional effort to explain or respond to abnormal changes in my training data.”

Items were rated from 1 (strongly disagree) to 5 (strongly agree), and the mean item score was used to represent DTMS. In the formal sample, corrected item–total correlations (CITCs) ranged from 0.644 to 0.688. Cronbach’s α decreased when any individual item was removed, indicating that no item warranted deletion on the basis of internal consistency. Confirmatory factor analysis showed standardized factor loadings ranging from 0.67 to 0.84, all of which were statistically significant (*p* < 0.001). The scale demonstrated satisfactory internal consistency and convergent validity (Cronbach’s α = 0.89, CR = 0.91, AVE = 0.57), supporting its use for assessing monitoring-related psychological pressure in data-intensive competitive training environments.

Evaluative anxiety was assessed as athletes’ anticipatory concerns about unfavorable judgments and performance-related consequences when confronted with training-data feedback, performance comparisons, and coaching evaluations. Measurement development drew on research concerning fear of negative evaluation and performance-related evaluative anxiety and was contextually adapted to data-driven competitive training environments (Watson and Friend, 1969; Leary, 1983; Cavanaugh et al., 2000). The adaptation retained the core conceptual elements of anticipated negative evaluation and evaluative concern while specifying the situational context in terms of training-data feedback, intra-team comparisons, anomalous indicators, and coaching judgments.

The final measure comprised six items. Example items included: “I worry that poor training data will negatively affect my coach’s evaluation of me,” “When monitoring data show abnormalities, I worry that others will perceive me as being in poor training condition,” and “I feel nervous when my training data are compared with those of my teammates.” Items were rated from 1 (strongly disagree) to 5 (strongly agree), and the mean item score represented the level of evaluative anxiety.

In the formal sample, CITCs for the six items ranged from 0.587 to 0.660, and removal of any individual item did not improve overall internal consistency. Confirmatory factor analysis yielded standardized factor loadings ranging from 0.65 to 0.83, all of which were statistically significant (*p* < 0.001). The scale demonstrated satisfactory internal consistency and convergent validity (Cronbach’s α = 0.84, CR = 0.88, AVE = 0.56), supporting its use for assessing athletes’ evaluative anxiety in data-driven training environments.

Psychological exhaustion was assessed as a state of depleted psychological resources associated with sustained exposure to monitoring-related demands and evaluative concerns. The construct was distinguished from general physical fatigue by emphasizing the subjective depletion of psychological energy associated with continued vigilance, data interpretation, self-regulation, and emotional control. Item development was informed by research on mental fatigue, self-regulatory depletion, and the Job Demands–Resources Model, with semantic adaptations made to competitive training contexts (Demerouti et al., 2001; Bakker and Demerouti, 2007; Marcora et al., 2009; Hagger et al., 2010).

The items primarily captured persistent mental fatigue, the additional psychological effort required to maintain concentration during training, and difficulty disengaging from monitoring-related concerns. This emphasis was intended to distinguish psychological resource depletion from general physical fatigue. The final measure comprised six items. Example items included: “I often feel mentally exhausted when faced with continuous training-data feedback,” “I need to exert considerable mental effort to maintain concentration during training,” and “I find it difficult to relax when I am constantly paying attention to training data.”

Items were rated from 1 (strongly disagree) to 5 (strongly agree), and the mean item score represented psychological exhaustion. In the formal sample, CITCs ranged from 0.629 to 0.668, and removal of any individual item did not improve overall internal consistency. Confirmatory factor analysis showed standardized factor loadings ranging from 0.68 to 0.85, all of which were statistically significant (*p* < 0.001). The scale demonstrated satisfactory internal consistency and convergent validity (Cronbach’s α = 0.86, CR = 0.90, AVE = 0.60), supporting its use for assessing psychological resource depletion in data-driven training environments.

Athlete burnout was assessed in terms of emotional and physical exhaustion, reduced athletic accomplishment, and sport devaluation under conditions of sustained training demands. Measurement was based on the Athlete Burnout Questionnaire (ABQ) developed by Raedeke and Smith and was translated, back-translated, and contextually adapted for Chinese competitive sport settings (Raedeke, 1997; Raedeke and Smith, 2001). The instrument comprises three dimensions–emotional and physical exhaustion, reduced sense of athletic accomplishment, and sport devaluation–with a total of 15 items.

Example items included: “I often feel physically and mentally exhausted from training,” “I feel that my progress in this sport is less apparent than it used to be,” and “I sometimes feel that continuing to participate in this sport has lost its meaning.” Items were rated from 1 (strongly disagree) to 5 (strongly agree). Items within the reduced-accomplishment dimension were scored in accordance with the scoring procedure adopted for the scale so that higher overall scores represented higher levels of athlete burnout.

In the formal sample, CITCs for the 15 items ranged from 0.607 to 0.678, and removal of any individual item did not increase the overall Cronbach’s α. Confirmatory factor analysis supported the three-dimensional structure, with standardized factor loadings ranging from 0.63 to 0.86, all of which were statistically significant (*p* < 0.001). The overall scale demonstrated high internal consistency (Cronbach’s α = 0.93, CR = 0.94, AVE = 0.49). Cronbach’s α values for the three subscales were 0.84, 0.82, and 0.86, respectively, indicating satisfactory reliability for the assessment of athlete burnout.

Gender and sport type were included as grouping variables in the multigroup analyses. Gender was self-reported by participants and coded in accordance with the specified grouping procedure. Sport type was classified by the research team on the basis of participants’ primary sport, training characteristics, predominant physiological and performance demands, and performance structure. Drawing on commonly used sport-classification principles in training science, sports were operationally categorized into three groups: physical endurance sports, skill-based sports, and competitive ball sports (Bompa and Haff, 2009).

Physical endurance sports primarily included middle- and long-distance running, swimming, cycling, and rowing, in which training management typically relies heavily on workload, physiological, and performance indicators. Skill-based sports primarily included gymnastics, diving, wushu, and rhythmic gymnastics, which place greater emphasis on movement quality, technical difficulty, and execution consistency. Competitive ball sports primarily included basketball, soccer, volleyball, badminton, and table tennis, in which performance reflects the combined influence of physical workload, technical execution, tactical demands, and competitive situations.

This operational classification was intended to capture meaningful differences in the intensity and evaluative role of data-driven monitoring across sport contexts while facilitating subsequent multigroup comparisons. It should not be interpreted as implying that all sports within a given category employ identical monitoring practices or assign the same evaluative weight to quantitative indicators. Meaningful variation may remain across individual sports within each category.

All established instruments derived from English-language sources underwent translation and back-translation procedures. Two researchers with backgrounds in sport psychology and proficiency in relevant Chinese and English terminology independently completed the forward translation and back-translation, respectively. The research team then compared the back-translated version with the original instrument and revised expressions that showed semantic inconsistency or inadequate contextual appropriateness for Chinese competitive training environments, with the aim of enhancing semantic equivalence and cultural appropriateness (Brislin, 1970).

For newly developed or substantially adapted items, content relevance and semantic appropriateness were evaluated primarily through expert review and pilot testing. Expert ratings of item relevance, clarity, and contextual applicability averaged above 4.40 on a 5-point scale across the measurement instruments, providing preliminary evidence of satisfactory content representation. Item analyses conducted using the formal sample further showed that CITCs for all retained items exceeded 0.50 and that removal of any single item did not improve the internal consistency of the corresponding measure. All retained items were therefore included in the formal analyses.

Psychometric evaluation using the formal sample examined standardized factor loadings, Cronbach’s α, composite reliability (CR), and average variance extracted (AVE) to assess factor quality, internal consistency, and convergent validity. Discriminant validity among the focal constructs was further evaluated using the Fornell–Larcker criterion and the heterotrait–monotrait ratio (HTMT). Detailed results are reported in the measurement model analysis below.

### Data analysis methods

This study primarily used SPSS 26.0 and AMOS 24.0 for data processing and statistical analysis. Before the main analyses, the raw data were screened for missing values and response quality. Questionnaires were excluded if they contained substantial missing data, excessive consecutive identical responses, implausibly short completion times, or clear inconsistencies between demographic information and training background. The distributional properties of the core variables were subsequently examined. The absolute skewness values for all focal variables were below 2, and the absolute kurtosis values were below 7, indicating no substantial departure from normality for maximum likelihood estimation in structural equation modeling. Descriptive statistics, including means, standard deviations, and Pearson correlation coefficients, were then calculated for data-driven training monitoring stress (DTMS), evaluative anxiety, psychological exhaustion, and athlete burnout to characterize the preliminary associations among the study variables.

Because all focal variables were assessed using athlete self-reports collected at a single time point, potential common method variance was addressed through both procedural remedies and statistical assessments. Procedurally, the questionnaires were completed anonymously, participants were informed that there were no correct or incorrect answers, and items measuring different constructs were presented in a mixed order to reduce potential social-desirability and consistency biases. Statistically, Harman’s single-factor test was first conducted using an unrotated exploratory factor analysis of all focal items. The first unrotated factor accounted for 31.86% of the total variance, below the commonly used 40% reference value, providing no indication that a single factor dominated the observed covariance structure. A single-factor confirmatory model was subsequently estimated in AMOS and compared with the hypothesized four-factor measurement model. The single-factor model exhibited poor fit (χ^2^/df = 5.10, CFI = 0.746, TLI = 0.730, RMSEA = 0.084, SRMR = 0.086), whereas the hypothesized four-factor model demonstrated substantially better fit (χ^2^/df = 1.04, CFI = 0.998, TLI = 0.998, RMSEA = 0.008, SRMR = 0.027). Taken together, these findings provided supplementary evidence against the presence of a dominant single-method factor. Nevertheless, Harman’s single-factor test and single-factor model comparisons have limited sensitivity for detecting common method variance. Because all core measures were self-reported and collected contemporaneously, the possibility of residual common method variance cannot be excluded. Accordingly, the structural associations should be interpreted with appropriate caution.

Confirmatory factor analysis was used to evaluate the measurement structure and the empirical distinctiveness of the focal constructs. The hypothesized four-factor model comprised DTMS, evaluative anxiety, psychological exhaustion, and athlete burnout. Each observed indicator was specified to load only on its theoretically designated latent construct, correlations among the latent constructs were freely estimated, and no cross-loadings or correlated item residuals were specified. To avoid improving model fit through *post hoc*, data-driven modifications, no residual covariances, cross-loadings, or additional paths were introduced on the basis of modification indices.

The hypothesized four-factor model was compared with theoretically relevant three-factor, two-factor, and single-factor alternatives. In the three-factor model, evaluative anxiety and psychological exhaustion were combined into a single latent construct, whereas DTMS and athlete burnout remained separate. In the two-factor model, DTMS, evaluative anxiety, and psychological exhaustion were combined into one latent construct, while athlete burnout remained distinct. In the single-factor model, all observed indicators were specified to load on a single latent factor. Model fit was evaluated using multiple indices, including χ^2^/df, the Comparative Fit Index (CFI), the Tucker–Lewis Index (TLI), the Root Mean Square Error of Approximation (RMSEA), and the Standardized Root Mean Square Residual (SRMR). As general reference criteria, χ^2^/df values below 3, CFI and TLI values above 0.90, and RMSEA and SRMR values below 0.08 were considered indicative of acceptable fit; CFI and TLI values approaching or exceeding 0.95 and RMSEA and SRMR values below approximately 0.06 were interpreted as evidence of good fit. To enhance reporting transparency, the χ^2^ statistic, degrees of freedom, associated *p*-value, standardized factor loadings, and latent-variable correlations were also considered rather than relying on any single fit index.

The hypothesized four-factor model demonstrated very good fit to the data, χ^2^(554) = 573.85, *p* = 0.271, χ^2^/df = 1.04, CFI = 0.998, TLI = 0.998, RMSEA = 0.008, and SRMR = 0.027. These indices indicated close statistical correspondence between the hypothesized measurement structure and the observed covariance matrix. However, model adequacy was evaluated in conjunction with theoretical interpretability, standardized factor loadings, latent-variable correlations, and comparisons with competing measurement models. Fit indices approaching their conventional optimum values were therefore not treated as sufficient evidence of construct validity in isolation.

Reliability and convergent validity were evaluated using standardized factor loadings, Cronbach’s α, composite reliability (CR), and average variance extracted (AVE). All standardized factor loadings were statistically significant, and most exceeded 0.60. Cronbach’s α values ranged from 0.84 to 0.93, CR values ranged from 0.88 to 0.94, and AVE values ranged from 0.49 to 0.60. Overall, these values indicated satisfactory internal consistency and convergent validity, although the lowest AVE value of 0.49 was marginally below the conventional 0.50 reference value and was therefore interpreted in conjunction with the corresponding factor loadings and CR. Discriminant validity was assessed using both the Fornell–Larcker criterion and the heterotrait–monotrait ratio (HTMT). According to the Fornell–Larcker criterion, the square root of each construct’s AVE should exceed its correlations with the other latent constructs. The results met this criterion for all focal variables, supporting the empirical distinctiveness of DTMS, evaluative anxiety, psychological exhaustion, and athlete burnout. Detailed HTMT results are reported in the measurement model analysis below.

Structural relationships were subsequently examined using structural equation modeling. In accordance with the hypothesized model, DTMS was specified as the predictor variable, evaluative anxiety and psychological exhaustion as sequential intervening variables, and athlete burnout as the outcome variable. Age, athletic level, years of sport-specific training, weekly training frequency, and team type were included as control variables to reduce potential confounding associated with demographic characteristics, training exposure, and organizational training context. Parameters were estimated using maximum likelihood estimation, and standardized path coefficients, standard errors, significance levels, and overall model-fit indices were reported.

Indirect associations were evaluated using bias-corrected nonparametric bootstrap procedures with 5,000 resamples and 95% confidence intervals. An indirect association was considered statistically significant when its confidence interval did not include zero. Three theoretically specified indirect pathways were examined: DTMS → evaluative anxiety → athlete burnout; DTMS → psychological exhaustion → athlete burnout; and DTMS → evaluative anxiety → psychological exhaustion → athlete burnout. The bootstrap procedure provided more robust inference for indirect associations without relying solely on normal-theory assumptions regarding their sampling distributions.

When both a direct association and the corresponding indirect association were statistically significant, the findings were interpreted as indicating the coexistence of direct and indirect statistical pathways. When the direct association was not statistically significant but the indirect association remained significant, the observed relationship was interpreted as operating primarily through the corresponding indirect pathway. These classifications were used solely to describe statistical patterns within the structural model and were not treated as evidence of temporal or causal mechanisms.

Because DTMS, evaluative anxiety, psychological exhaustion, and athlete burnout were measured concurrently using cross-sectional data, the directional paths specified in the structural model reflect theoretically derived associations rather than empirically established temporal sequences. Accordingly, the pathway DTMS → evaluative anxiety → psychological exhaustion → athlete burnout should be interpreted as a theoretically grounded associative structure consistent with the observed covariance pattern. Bootstrap indirect effects likewise represent statistical indirect associations and do not establish causal mediation. Reverse associations, reciprocal relationships, or alternative temporal sequences cannot be ruled out by the present research design.

Multigroup structural equation modeling was used to examine whether the DTMS–evaluative anxiety pathway differed by gender and sport type. Gender groups comprised male and female athletes, whereas sport type was categorized into physical endurance sports, skill-based sports, and competitive ball sports. Before structural paths were compared across groups, measurement invariance was evaluated sequentially at the configural, metric, and scalar levels. Configural invariance assessed whether the same factor structure was supported across groups; metric invariance constrained factor loadings to equality; and scalar invariance additionally constrained item intercepts. Changes in model fit were evaluated primarily using ΔCFI and ΔRMSEA, with ΔCFI ≤ 0.010 and ΔRMSEA ≤ 0.015 taken as evidence that the additional invariance constraints did not meaningfully worsen model fit.

Once an adequate level of measurement invariance had been established, structural path differences were examined by comparing an unconstrained structural model with models in which the focal DTMS–evaluative anxiety path was constrained to equality across groups. Chi-square difference tests, changes in CFI, and group-specific standardized path coefficients were jointly considered when evaluating between-group differences. The focal comparisons examined whether the association between DTMS and evaluative anxiety differed between male and female athletes and across the three sport-type groups.

All statistical tests were two-tailed, with the significance level set at α = 0.05. The results are reported in the following sequence: descriptive statistics and bivariate correlations, assessment of common method variance, measurement-model fit and alternative-model comparisons, reliability and validity analyses, structural path estimates, bootstrap confidence intervals for indirect associations, and multigroup comparisons by gender and sport type. Together, these analyses were used to examine the direct associations between DTMS and athlete burnout, the indirect pathways involving evaluative anxiety and psychological exhaustion, and group differences in the focal DTMS–evaluative anxiety relationship.

## Research findings

### Descriptive statistics and correlation analysis

To characterize the distributions of the core variables and their preliminary associations, descriptive statistics and Pearson correlation analyses were conducted for data-driven training monitoring stress (DTMS), evaluative anxiety, psychological exhaustion, and athlete burnout (see Figure 2 and Table 1). As shown in Table 1, the mean scores for all four constructs were slightly above the midpoint of the 5-point response scale, with sufficient variability across participants to support subsequent analyses.

**FIGURE 2:**
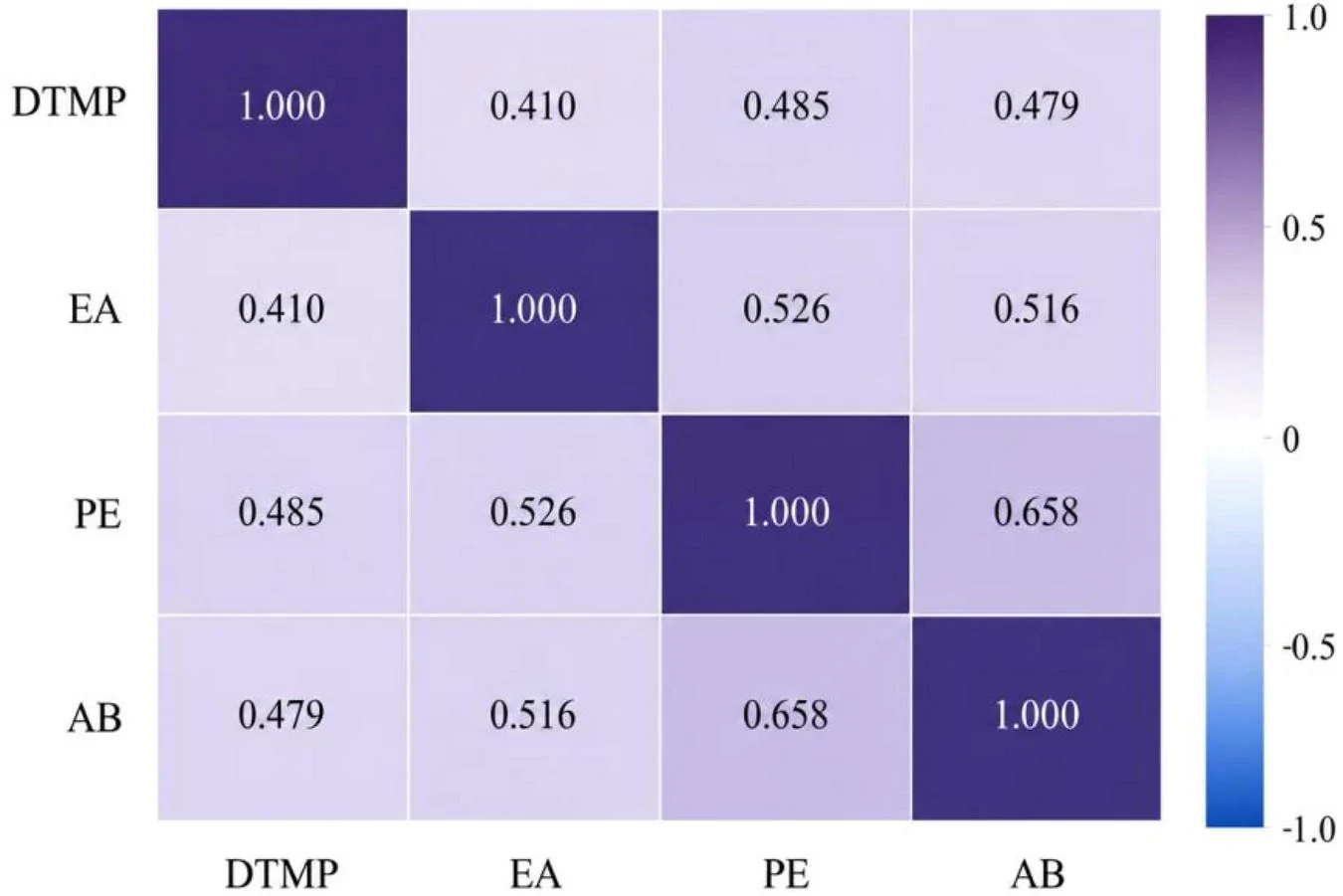
Pearson correlation matrix among core variables. DTMP, digital training monitoring pressure; EA, evaluative anxiety; PE, psychological exhaustion; AB, athlete burnout.

**TABLE 1:** Descriptive statistics.

Variable	Mean	Standard deviation
Digital training monitoring pressure (DTMP)	3.16	0.80
Evaluative anxiety (EA)	3.11	0.80
Psychological exhaustion (PE)	3.07	0.82
Athlete burnout (AB)	3.04	0.74

The mean DTMS score was 3.16 (SD = 0.80), indicating that participants reported a moderate level of psychological pressure associated with continuous data collection, metric comparison, and evaluative feedback. Evaluative anxiety had a mean score of 3.11 (SD = 0.80), suggesting a moderate level of concern about unfavorable evaluations in the context of training-data feedback, coaching judgments, and intra-team comparisons. The mean score for psychological exhaustion was 3.07 (SD = 0.82), indicating that participants reported a moderate degree of psychological resource depletion. Athlete burnout had a mean score of 3.04 (SD = 0.74), indicating a moderate level of burnout symptoms in the sample. Overall, the descriptive statistics revealed meaningful interindividual variation across the four constructs.

Pearson correlation coefficients are presented in Figure 2. DTMS was significantly and positively correlated with evaluative anxiety (*r* = 0.410, *p* < 0.001), indicating that higher levels of monitoring-related stress were associated with greater evaluative anxiety. DTMS was also significantly and positively correlated with psychological exhaustion (*r* = 0.485, *p* < 0.001) and athlete burnout (*r* = 0.479, *p* < 0.001), indicating that greater monitoring-related stress was associated with higher levels of psychological exhaustion and burnout.

Evaluative anxiety was significantly and positively correlated with psychological exhaustion (*r* = 0.526, *p* < 0.001) and athlete burnout (*r* = 0.516, *p* < 0.001). Thus, athletes reporting higher levels of evaluative anxiety also tended to report greater psychological exhaustion and more pronounced burnout symptoms. Psychological exhaustion showed the strongest bivariate association with athlete burnout (*r* = 0.658, *p* < 0.001), indicating that higher levels of psychological resource depletion were closely associated with higher levels of burnout. Overall, the direction and magnitude of the correlations were consistent with the hypothesized relational structure and provided a basis for subsequent structural model testing.

### Measurement model validation: confirmatory factor analysis (CFA)

To evaluate the measurement quality of the four latent constructs–data-driven training monitoring stress (DTMS), evaluative anxiety (EA), psychological exhaustion (PE), and athlete burnout (AB)–internal consistency, composite reliability, and convergent validity were examined. The results are presented in Table 2.

**TABLE 2:** Confirmatory factor analysis results.

Latent variable	Cronbach α	CR	AVE
DTMP	0.89	0.91	0.57
EA	0.84	0.88	0.56
PE	0.86	0.90	0.60
AB	0.93	0.94	0.49

As shown in Table 2, Cronbach’s α values were 0.89 for DTMS, 0.84 for EA, 0.86 for PE, and 0.93 for AB. All coefficients exceeded 0.80, indicating satisfactory internal consistency across the four measures. Composite reliability (CR) values were 0.91, 0.88, 0.90, and 0.94, respectively, all exceeding the commonly recommended criterion of 0.70. These results indicate that the indicators associated with each construct demonstrated satisfactory reliability.

With respect to convergent validity, the average variance extracted (AVE) values for DTMS, EA, and PE were 0.57, 0.56, and 0.60, respectively, all exceeding the conventional reference value of 0.50. The AVE for AB was 0.49, marginally below this reference value. However, AB demonstrated high internal consistency (Cronbach’s α = 0.93) and composite reliability (CR = 0.94), and its standardized factor loadings were generally satisfactory. Given that the AVE was only marginally below 0.50 and that the remaining reliability and measurement indicators were adequate, the convergent validity of AB was considered acceptable when evaluated in conjunction with these complementary criteria. Overall, the four measures demonstrated satisfactory reliability and broadly acceptable convergent validity, providing an adequate basis for subsequent measurement and structural model analyses.

Confirmatory factor analysis was subsequently conducted to examine the fit and empirical distinctiveness of the hypothesized four-factor measurement structure. In the theoretical model, each observed indicator was specified to load only on its designated latent construct, while correlations among the four latent constructs were freely estimated. To assess whether the constructs could be empirically distinguished, the hypothesized four-factor model was compared with theoretically relevant three-factor, two-factor, and single-factor alternatives. In the three-factor model, EA and PE were combined into a single latent factor, while DTMS and AB remained separate. In the two-factor model, DTMS, EA, and PE were combined into one latent factor, with AB retained as a separate factor. In the single-factor model, all observed indicators were specified to load on a common latent factor. The results of these model comparisons are presented in Table 3.

**TABLE 3:** Measurement model and alternative model comparison.

Model	χ^2^	df	*p*	χ^2^/df	CFI	TLI	RMSEA	SRMR
Four-factor model	573.85	554	0.271	1.04	0.998	0.998	0.008	0.027
Three-factor model (EA + PE)	1071.03	557	<0.001	1.92	0.943	0.939	0.040	0.043
Two-factor model (DTMP + EA + PE)	2037.76	559	<0.001	3.65	0.837	0.826	0.067	0.071
Single-factor model	2858.22	560	<0.001	5.10	0.746	0.730	0.084	0.086

The hypothesized four-factor model demonstrated very good fit to the data, χ^2^(554) = 573.85, *p* = 0.271, χ^2^/df = 1.04, CFI = 0.998, TLI = 0.998, RMSEA = 0.008, and SRMR = 0.027. By comparison, the three-factor, two-factor, and single-factor models exhibited progressively poorer fit after theoretically distinct constructs were combined, with the deterioration being particularly pronounced for the two-factor and single-factor models. These comparisons indicate that the four-factor structure provided a substantially better representation of the observed measurement relationships among DTMS, EA, PE, and AB. The findings therefore provide additional empirical support for treating the four constructs as related but distinguishable components of the proposed theoretical model.

Because several fit indices for the four-factor model approached their conventional optimum values, model adequacy was not evaluated solely on the basis of individual fit statistics. Instead, the interpretation incorporated the prespecified measurement structure, standardized factor loadings, latent-variable correlations, and comparisons with competing models. Accordingly, the excellent global fit was interpreted as evidence of close correspondence between the hypothesized covariance structure and the observed data rather than as sufficient evidence of measurement validity in isolation.

The latent-variable correlations provided further evidence regarding construct distinctiveness. DTMS was correlated with EA, PE, and AB at 0.475, 0.553, and 0.530, respectively. EA was correlated with PE at 0.617 and with AB at 0.584, while the correlation between PE and AB was the highest, at 0.738. All latent-variable correlations remained below 0.85, suggesting that none of the construct pairs exhibited an excessively high degree of empirical overlap. In particular, the correlation between DTMS and EA (0.475) indicates that, although both constructs are situated within data-driven evaluative training contexts, they remain empirically distinguishable. The relatively stronger association between PE and AB (0.738) is consistent with their conceptual proximity, as both involve experiences related to exhaustion. Nevertheless, the magnitude of this correlation remained below commonly used thresholds for problematic construct overlap. Conceptually, PE primarily reflects psychological resource depletion, whereas AB represents a broader multidimensional syndrome encompassing emotional and physical exhaustion, reduced athletic accomplishment, and sport devaluation.

Discriminant validity was further evaluated using the Fornell–Larcker criterion and the heterotrait–monotrait ratio (HTMT). For each construct, the square root of the AVE exceeded its correlations with the other latent constructs, satisfying the Fornell–Larcker criterion. HTMT values ranged from 0.474 to 0.737, all below the more conservative reference value of 0.85. Taken together, the latent-variable correlations, Fornell–Larcker results, HTMT values, and competing-model comparisons provided convergent evidence of acceptable discriminant validity among DTMS, EA, PE, and AB. These findings support treating the four variables as theoretically related yet empirically distinguishable constructs. However, statistical distinctiveness should not be interpreted as implying the complete absence of conceptual overlap between theoretically adjacent constructs.

Because all focal variables were assessed through athlete self-reports collected at a single time point, the potential influence of common method variance was also examined. Harman’s single-factor test showed that the first unrotated factor accounted for 31.86% of the total variance, below the commonly used 40% reference value. In addition, the single-factor measurement model exhibited substantially poorer fit than the hypothesized four-factor model. Together, these findings provided no clear indication that a single common factor dominated the observed covariance structure.

Nevertheless, these results do not rule out common method variance. Harman’s single-factor test and single-factor model comparisons have limited sensitivity for detecting method effects, and all focal constructs in the present study were measured using contemporaneous self-report data. Residual common method variance may therefore remain and may have influenced the observed associations among the variables. Accordingly, the subsequent structural paths and indirect associations should be interpreted with appropriate caution.

### Structural path analysis

Building on the satisfactory fit of the measurement model, a structural equation model was estimated to examine the structural associations among data-driven training monitoring stress (DTMS), evaluative anxiety, psychological exhaustion, and athlete burnout. DTMS was specified as the exogenous variable, evaluative anxiety and psychological exhaustion as sequential intervening variables, and athlete burnout as the outcome variable. Age, athletic level, years of sport-specific training, weekly training frequency, and team type were included as control variables. The standardized path coefficients are presented in Figure 3.

**FIGURE 3:**
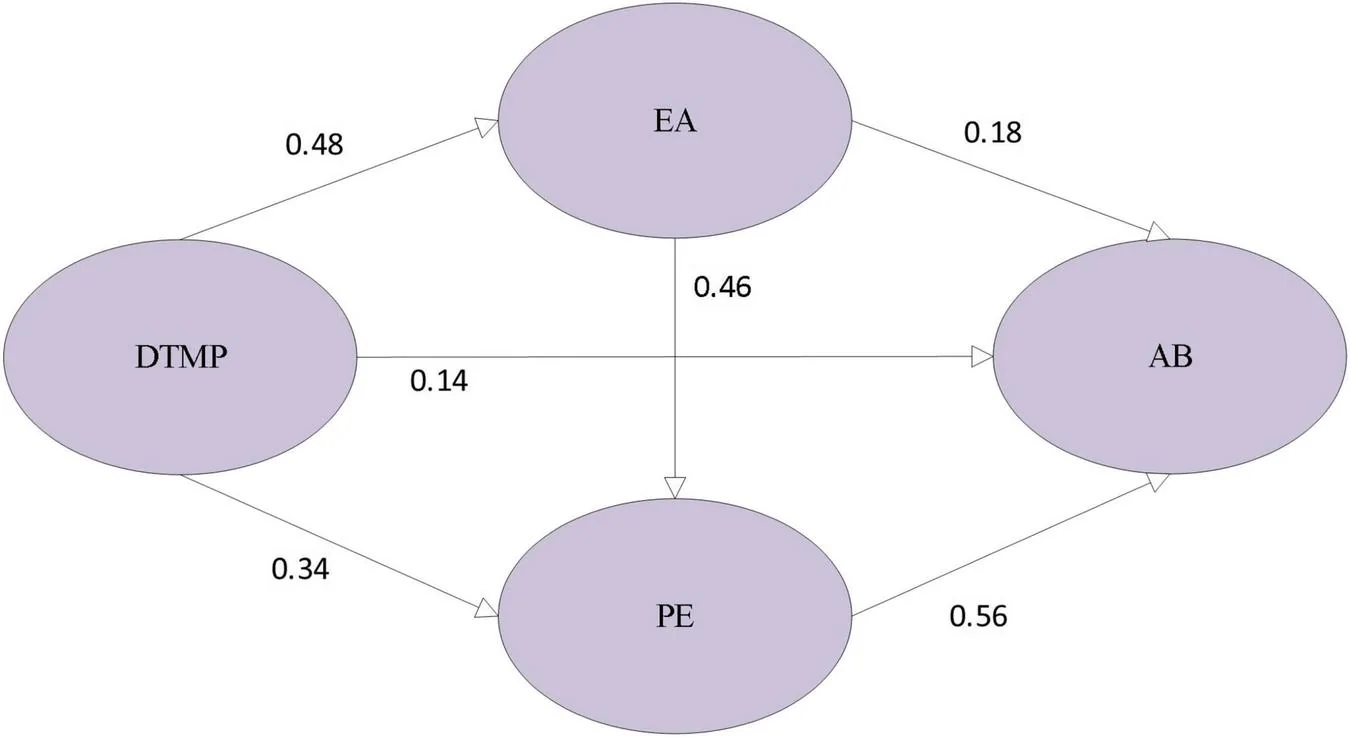
Standardized path coefficients in the SEM.

The results showed that DTMS was significantly and positively associated with evaluative anxiety (β = 0.48, *p* < 0.001) and psychological exhaustion (β = 0.34, *p* < 0.001). Thus, higher levels of DTMS were associated with greater evaluative anxiety and psychological exhaustion. The direct association between DTMS and athlete burnout also remained statistically significant after evaluative anxiety and psychological exhaustion were included in the model (β = 0.14, *p* < 0.001), indicating that DTMS retained a significant direct association with burnout beyond the specified indirect pathways.

Regarding the relationships among the intervening variables and athlete burnout, evaluative anxiety was significantly and positively associated with psychological exhaustion (β = 0.46, *p* < 0.001) and athlete burnout (β = 0.18, *p* < 0.001). Psychological exhaustion was also significantly and positively associated with athlete burnout (β = 0.55, *p* < 0.001), representing the strongest structural association with burnout among the psychological variables included in the model. These findings indicate that higher levels of evaluative anxiety were associated with greater psychological exhaustion and that greater psychological exhaustion was, in turn, associated with more pronounced athlete burnout.

Indirect associations were examined using bias-corrected bootstrap procedures with 5,000 resamples and 95% confidence intervals. The indirect association between DTMS and athlete burnout through evaluative anxiety was statistically significant (β = 0.082, 95% CI [0.051, 0.115]). The indirect association through psychological exhaustion was also statistically significant (β = 0.152, 95% CI [0.110, 0.195]). In addition, the sequential indirect association through evaluative anxiety and psychological exhaustion was statistically significant (β = 0.076, 95% CI [0.055, 0.101]). The total indirect association was β = 0.310, 95% CI [0.261, 0.360]. None of the corresponding confidence intervals included zero. Taken together, these results support significant indirect associations between DTMS and athlete burnout through evaluative anxiety and psychological exhaustion separately, as well as a significant sequential indirect pathway involving evaluative anxiety followed by psychological exhaustion.

### Moderating effects analysis

Building on the structural model results, multigroup structural equation modeling was conducted to examine whether the focal structural pathway differed by gender and sport type. Consistent with the study hypotheses, the multigroup analyses focused specifically on the association between data-driven training monitoring stress (DTMS) and evaluative anxiety (EA). Before comparing structural paths across groups, measurement invariance was assessed separately for gender and sport type at the configural, metric, and scalar levels to determine whether DTMS, EA, psychological exhaustion (PE), and athlete burnout (AB) were measured comparably across groups. The results of the measurement invariance tests are presented in Table 4.

**TABLE 4:** Measurement invariance test results.

Grouping variable	Model	CFI	TLI	RMSEA	Δ CFI	Δ RMSEA	Δχ^2^(df)	*p*
Gender	Configural	0.993	0.992	0.010	–	–	–	–
Gender	Metric	0.992	0.992	0.010	−0.0003	0.0001	33.86 (31)	0.331
Gender	Scalar	0.992	0.992	0.010	<0.0001	−0.0002	30.77 (31)	0.478
Sport type	Configural	0.981	0.980	0.013	–	–	–	–
Sport type	Metric	0.980	0.979	0.013	−0.0012	0.0002	72.65 (62)	0.167
Sport type	Scalar	0.981	0.981	0.013	0.0014	−0.0007	49.22 (62)	0.880

As shown in Table 4, the configural invariance model for gender demonstrated good fit, indicating that the same general factor structure was supported for male and female athletes. When factor loadings were constrained to equality across gender groups, model fit changed only minimally (ΔCFI = −0.0003, ΔRMSEA = 0.0001), and the chi-square difference test was nonsignificant, Δχ^2^(31) = 33.86, *p* = 0.331. These results supported metric invariance. Additional constraints on item intercepts likewise produced no meaningful deterioration in model fit (ΔCFI < 0.0001, ΔRMSEA = −0.0002), and the chi-square difference test remained nonsignificant, Δχ^2^(31) = 30.77, *p* = 0.478, supporting scalar invariance.

A similar pattern was observed across sport-type groups. Relative to the configural model, the metric invariance model showed only minor changes in fit (ΔCFI = −0.0012, ΔRMSEA = 0.0002), and the chi-square difference test was nonsignificant, Δχ^2^(62) = 72.65, *p* = 0.167. Likewise, constraining item intercepts to equality resulted in minimal changes in model fit (ΔCFI = −0.0007, ΔRMSEA = 0.0002), with a nonsignificant chi-square difference, Δχ^2^(62) = 49.22, *p* = 0.880. Because the changes in CFI and RMSEA remained within the prespecified criteria, configural, metric, and scalar invariance were considered acceptable across both gender and sport-type groups. These findings provided an adequate measurement basis for subsequent comparisons of structural paths across groups.

After measurement invariance had been established, unconstrained structural models were compared with models in which the focal DTMS–EA pathway was constrained to equality across groups. Chi-square difference tests were used to determine whether imposing equality constraints significantly worsened model fit. The results are presented in Table 5.

**TABLE 5:** Multi-group SEM results.

Grouping variable	Group	Path	Standardized coefficient (β)	Δχ^2^ (df = 1)	Significance
Gender	Male vs. female	DTMP → EA	0.34 VS. 0.61	5.44	*p* = 0.020, significant
Sport type	Endurance vs. skill	DTMP → EA	0.59 VS. 0.31	7.99	*p* = 0.005, significant
Sport type	Endurance vs. ball	DTMP → EA	0.59 VS. 0.47	1.78	*p* = 0.182, n.s
Sport type	Ball vs. skill	DTMP → EA	0.47 VS. 0.31	2.13	*p* = 0.145, n.s.

For gender, the DTMS–EA pathway differed significantly between male and female athletes. Constraining this path to equality across the two groups produced a significant deterioration in model fit, Δχ^2^(1) = 5.44, *p* = 0.020. The standardized path coefficient was higher among female athletes (β = 0.61) than among male athletes (β = 0.34), indicating that the association between DTMS and evaluative anxiety was stronger in the female group. Within the present sample, therefore, higher levels of monitoring-related stress were more strongly associated with evaluative anxiety among female athletes than among male athletes. Although this difference may reflect group variation in the perceived consequences of training-data evaluation, judgments of current performance status, or competition-related opportunities, the present cross-sectional design does not permit direct identification of the psychological mechanisms underlying this difference.

Differences in the DTMS–EA pathway were also examined across sport types. The contrast between physical endurance sports and skill-based sports was statistically significant. The standardized path coefficient was higher for physical endurance sports (β = 0.59) than for skill-based sports (β = 0.31), and constraining the pathway to equality significantly worsened model fit, Δχ^2^(1) = 7.99, *p* = 0.005. These findings indicate that the association between DTMS and evaluative anxiety was stronger among athletes in physical endurance sports than among those in skill-based sports.

One possible contextual explanation is that physical endurance sports rely more heavily on continuously quantified indicators such as heart rate, distance, speed, training load, and recovery status. These indicators are often directly incorporated into assessments of competitive condition, training adjustments, and within-team comparisons, potentially increasing the evaluative salience of monitoring data. In skill-based sports, by contrast, performance evaluation tends to depend more strongly on movement quality, technical execution, difficulty, and consistency, with individual quantitative monitoring indicators often playing a supplementary role. These sport-specific characteristics may help contextualize the stronger DTMS–EA association observed in physical endurance sports, although they should not be interpreted as causal mechanisms established by the present study.

The remaining pairwise comparisons were not statistically significant. The DTMS–EA pathway did not differ significantly between physical endurance sports and competitive ball sports (β = 0.59 vs. β = 0.47), Δχ^2^(1) = 1.78, *p* = 0.182, or between competitive ball sports and skill-based sports (β = 0.47 vs. β = 0.31), Δχ^2^(1) = 2.13, *p* = 0.145. Although the standardized coefficients showed a descriptive ordering–highest for physical endurance sports, intermediate for competitive ball sports, and lowest for skill-based sports–this pattern should not be interpreted as evidence of systematic differences across all sport types.

The intermediate coefficient observed for competitive ball sports may reflect the more complex role of monitoring data in these settings. Performance in ball sports is influenced not only by physical workload, speed variation, and other physiological indicators but also by tactical execution, teamwork, positional demands, and in-game decision-making. Consequently, the evaluative significance of monitoring information may differ from that observed in both physical endurance and skill-based sports. However, because the relevant pairwise differences were not statistically significant, this intermediate pattern should be regarded as descriptive rather than as evidence of a stable sport-type effect.

## Discussion

### Data-driven training monitoring stress as an emerging contextual antecedent of athlete burnout

The structural equation modeling results indicated a significant positive association between data-driven training monitoring stress (DTMS) and athlete burnout (β = 0.14, *p* < 0.001). This association remained statistically significant after evaluative anxiety and psychological exhaustion were included in the model, indicating that DTMS was associated with athlete burnout not only through the specified indirect pathways but also through a residual direct pathway. This finding suggests that research on training monitoring technologies should extend beyond their functional contributions to training-load assessment, fatigue detection, and injury-risk management to consider the psychological experiences that may accompany the integration of data-driven monitoring into competitive training environments and their potential relevance to athlete burnout.

From the perspective of the Technology Stress Generators Model, technology-related stress may arise when technological systems continuously reshape task structures, information-processing demands, and evaluative environments (Tarafdar et al., 2007; Ragu-Nathan et al., 2008). When data-driven monitoring becomes embedded in competitive training, athletes’ physical condition, training load, recovery status, and performance fluctuations are increasingly transformed into continuously visible, comparable, and traceable indicators. Consequently, athletes’ bodily sensations and situational training performance are no longer interpreted solely through the experiential judgments of athletes and coaches but are increasingly incorporated into systematic processes of data recording, comparison, and evaluation. In this sense, monitoring technologies may alter the structure of performance visibility within training environments by making athletes’ physical and performance states more continuously observable and subject to interpretation.

These characteristics provide an important contextual basis for understanding the association between DTMS and athlete burnout. The Challenge–Hindrance Stressor Framework proposes that the psychological implications of stressors depend partly on how individuals appraise their relevance to goal attainment and their potential consequences (Cavanaugh et al., 2000; LePine et al., 2005). When monitoring demands are perceived as facilitating competence development, training adjustment, and goal attainment, they may acquire challenge-related characteristics. In contrast, when monitoring outcomes are perceived as constraining development, reducing control, or threatening valued opportunities, the same demands may acquire hindrance characteristics. In training practice, monitoring data may serve a constructive function when they are primarily used to help athletes understand training responses, regulate recovery, and optimize training programs. However, when monitoring outcomes are closely tied to team rankings, competition selection, increased training demands, attribution of responsibility, or coaching evaluations, monitoring may extend beyond technical feedback and acquire greater evaluative significance for athletes.

This pattern of findings extends athlete burnout research by highlighting the relevance of increasingly data-intensive training environments. Previous studies have largely examined burnout in relation to sustained training demands, inadequate recovery, reduced perceptions of accomplishment, and sport devaluation (Raedeke, 1997; Raedeke and Smith, 2001; Gustafsson et al., 2017). The present findings further show that higher levels of DTMS are associated with higher levels of athlete burnout, suggesting that the evaluative environment created by continuous digital monitoring may represent an additional contextual factor warranting attention alongside conventional training demands. Even when athletes continue to fulfill prescribed training requirements, repeated exposure to quantified performance indicators, comparative feedback, and the interpretation of monitoring outcomes may be associated with additional psychological burden and a reduced sense of control over the training experience. From this perspective, data-driven monitoring may not necessarily increase the objective volume of training itself; rather, it may be associated with additional psychological demands arising from the need to remain continuously measurable, interpretable, and evaluable within the training system.

### Indirect association pathway through evaluative anxiety

Structural path analysis and bootstrap testing showed that data-driven training monitoring stress (DTMS) was significantly and positively associated with evaluative anxiety (β = 0.48, *p* < 0.001), which was, in turn, significantly associated with athlete burnout (β = 0.18, *p* < 0.001). The indirect association between DTMS and athlete burnout through evaluative anxiety was also statistically significant (β = 0.082, 95% CI [0.051, 0.115]). These findings suggest that evaluative anxiety may represent an important psychological mechanism for understanding the association between DTMS and athlete burnout.

Evaluative anxiety highlights that the psychological significance of data-driven monitoring may lie not only in continuous monitoring itself but also in the evaluative implications attached to it. Research on fear of negative evaluation suggests that concerns about unfavorable judgments from others may be associated with heightened tension, avoidance tendencies, and self-protective responses in evaluative social contexts (Watson and Friend, 1969; Leary, 1983). In competitive training environments, data-driven monitoring continuously renders athletes’ physical condition, responses to training load, and training outcomes visible through quantitative indicators. In doing so, complex and context-dependent training experiences may be translated into standardized metrics that are more readily compared and evaluated. When such metrics are incorporated into coaching judgments, team rankings, training adjustments, or competition opportunities, athletes are confronted not only with performance information but also with the potential evaluative consequences associated with that information. From this perspective, athletes who interpret monitoring data as salient signals of judgment may be more likely to experience evaluative anxiety. Evaluative anxiety therefore provides a plausible psychological pathway through which DTMS may be associated with athlete burnout.

These findings are also consistent with the Challenge–Hindrance Stressor Framework. Data-driven monitoring may acquire challenge-related characteristics when it provides interpretable information that supports training adjustment, workload regulation, and recovery optimization. Its psychological implications, however, depend partly on how athletes appraise the purpose and consequences of the feedback. When monitoring data are used primarily to clarify physiological responses and support performance development, athletes may regard them as informative resources for adaptation. By contrast, when monitoring outcomes are persistently used for comparison, selection, sanction, or attribution of responsibility, the same demands may acquire hindrance characteristics (Cavanaugh et al., 2000; LePine et al., 2005). The significant association between DTMS and evaluative anxiety observed in the present study is consistent with the possibility that monitoring becomes more psychologically demanding when athletes perceive data feedback as carrying substantial evaluative consequences. Nevertheless, this interpretation represents a theoretically informed explanation of cross-sectional associations rather than a directly established causal mechanism.

From the perspective of athlete burnout, evaluative anxiety may also be relevant because it can shape how athletes experience and interpret their training environment. Under intensive monitoring, athletes may repeatedly attend to whether their indicators meet expected standards, whether short-term fluctuations will be interpreted negatively, and whether monitoring outcomes will influence coaching judgments or valued opportunities. Such persistent evaluative vigilance may be associated with reduced perceptions of autonomy and accomplishment, as well as a stronger orientation toward avoiding unfavorable outcomes. Under these conditions, training may increasingly be experienced as a process of demonstrating adequacy and managing evaluation rather than solely as an opportunity for performance development. Sustained exposure to such evaluative demands may therefore coincide with greater emotional exhaustion, diminished meaning attached to training, and stronger sport-related detachment.

Previous research has emphasized that athlete burnout is associated not only with accumulated training demands but also with prolonged psychological stress, reduced athletic accomplishment, and sport devaluation (Raedeke, 1997; Raedeke and Smith, 2001; Gustafsson et al., 2017). The present findings extend this perspective by indicating that evaluative anxiety within data-intensive training environments may constitute an important psychological pathway linking monitoring-related stress to burnout. Accordingly, the psychological consequences of data-driven training monitoring may depend not only on the quantity and frequency of data collection but also on the extent to which athletes perceive monitoring information as carrying consequential judgments about their competence, status, and future opportunities.

### Resource-depletion association of psychological exhaustion

Structural path analysis showed that evaluative anxiety was significantly and positively associated with psychological exhaustion (β = 0.46, *p* < 0.001), while psychological exhaustion exhibited a strong positive association with athlete burnout (β = 0.55, *p* < 0.001). Bootstrap analyses further showed that the indirect association between data-driven training monitoring stress (DTMS) and athlete burnout through psychological exhaustion was statistically significant (β = 0.152, 95% CI [0.110, 0.195]). The sequential indirect association through evaluative anxiety and psychological exhaustion was also statistically significant (β = 0.076, 95% CI [0.055, 0.101]). Taken together, these findings suggest that psychological exhaustion may represent an important resource-depletion mechanism for understanding the associations among DTMS, evaluative anxiety, and athlete burnout.

This pattern of findings is consistent with the Job Demands–Resources (JD–R) Model. The JD–R framework proposes that sustained exposure to high demands, particularly when adequate resources are unavailable, is associated with progressive resource depletion, exhaustion, and burnout (Demerouti et al., 2001; Bakker and Demerouti, 2007). Data-driven training monitoring is not equivalent to training load itself; rather, it may introduce additional psychological demands alongside athletes’ existing physical and performance demands. Athletes must not only complete prescribed training tasks but also interpret fluctuations in monitoring indicators, respond to repeated feedback, maintain performance under continuous evaluation, and account for anomalous results when monitoring data deviate from expectations. These recurring requirements may place additional demands on attentional, emotion-regulation, and self-regulatory resources. From this perspective, psychological exhaustion provides a theoretically plausible resource-depletion pathway through which monitoring-related stress may be associated with athlete burnout.

The findings further suggest that the association between DTMS and psychological exhaustion may be understood in terms of the cumulative demands associated with repeated monitoring, although the cross-sectional design does not permit direct examination of such a temporal process. Athletes may not immediately exhibit resistance or disengagement in response to monitoring; nevertheless, sustained exposure to performance indicators, comparative feedback, and evaluative expectations may be associated with fewer psychological resources being available to meet subsequent training demands. Research on mental fatigue has similarly indicated that prolonged cognitive effort may be associated with greater subjective fatigue and a diminished capacity to sustain performance on subsequent tasks (Marcora et al., 2009; Hagger et al., 2010). In competitive training environments, athletes exposed to frequent feedback, persistent comparison, and repeated interpretation of anomalous indicators may therefore report greater psychological exhaustion, difficulty sustaining concentration, and impaired psychological recovery, even in the absence of substantial increases in physical training load. Accordingly, burnout in data-intensive training environments may be associated not only with the magnitude of physical training demands but also with the balance between additional psychological demands and the resources available to manage them.

The sequential indirect association further revealed a statistical pattern consistent with the theoretically proposed progression from evaluative response to resource depletion and, subsequently, to burnout. Evaluative anxiety reflects athletes’ concerns about the potential consequences associated with monitoring outcomes, whereas psychological exhaustion captures the depletion of psychological resources associated with sustained evaluative demands. When athletes repeatedly worry that their monitoring data may influence coaching judgments, team status, or competition opportunities, greater attentional and self-regulatory resources may be devoted to interpreting feedback, managing emotional responses, and maintaining a favorable performance image. Higher levels of psychological exhaustion are, in turn, associated with more pronounced athlete burnout. Previous research has identified exhaustion as a central component of athlete burnout and has linked burnout more broadly to reduced athletic accomplishment and sport devaluation (Raedeke and Smith, 2001; Gustafsson et al., 2017). The present findings extend this perspective by suggesting that, within data-intensive training environments, psychological exhaustion may constitute an important resource-depletion pathway linking monitoring-related evaluative demands to athlete burnout. Nevertheless, the observed sequential association should be interpreted as theoretically consistent with the proposed mechanism rather than as evidence of an established temporal or causal sequence.

### Gender and sport type as boundary conditions in the transformation of monitoring stress

Multigroup structural equation modeling revealed a significant gender difference in the association between data-driven training monitoring stress (DTMS) and evaluative anxiety, Δχ^2^(1) = 5.44, *p* = 0.020. Specifically, the standardized path coefficient was higher among female athletes (β = 0.61) than among male athletes (β = 0.34), indicating that DTMS was more strongly associated with evaluative anxiety in the female group within the present sample. This finding should not be interpreted as evidence of a stable gender difference in dispositional anxiety. Rather, it suggests that the psychological significance of monitoring-related stress may vary according to athletes’ evaluative experiences, perceived performance consequences, and self-presentational demands.

Research on fear of negative evaluation indicates that, in high-stakes evaluative contexts, heightened concern about external judgments and their potential consequences may be associated with greater tension and self-protective responses (Watson and Friend, 1969; Leary, 1983). From this perspective, the observed gender difference may be better understood in relation to the evaluative contexts in which athletes operate and the ways in which they interpret the consequences of monitoring outcomes, rather than as reflecting an inherent tendency toward greater anxiety among female athletes. Data-driven monitoring continuously externalizes athletes’ physical condition and training performance through visible and comparable indicators. When such indicators are linked to judgments of ability, within-team comparisons, or the allocation of valued opportunities, their evaluative significance may become particularly salient. Female athletes in the present sample may therefore have been more sensitive to the evaluative implications of fluctuations in monitoring data, contributing to the stronger DTMS–evaluative anxiety association observed in this group. However, the study did not directly assess gendered evaluation experiences, self-presentational demands, or perceived physical-evaluation pressure. These explanations should therefore be regarded as theoretically informed interpretations that require direct examination in future research.

The observed gender difference further suggests that ostensibly uniform monitoring systems may not be experienced in psychologically equivalent ways across athlete groups. Although data-driven monitoring presents standardized indicators and apparently objective criteria, the meaning attached to those indicators may vary according to athletes’ prior evaluative experiences and perceptions of their consequences. When monitoring results are directly incorporated into ranking, accountability, selection, or opportunity allocation, quantitatively standardized systems may nevertheless be associated with unequal levels of perceived evaluative pressure. The relevant question is therefore not simply whether female athletes are more susceptible to anxiety, but whether identical monitoring information acquires different levels of perceived threat across distinct evaluative and social contexts. From a practical perspective, this finding suggests that data feedback should minimize categorical judgments based on isolated results and instead provide contextualized explanations of training-load variation, recovery status, and phase-specific fluctuations, particularly for athletes who exhibit greater sensitivity to evaluative feedback.

Sport type was also associated with differences in the DTMS–evaluative anxiety pathway. The sport categories used in this study should be understood as an operational classification of data-driven training contexts rather than as a general taxonomy of competitive sport. The classification was based on the extent of reliance on quantitative monitoring indicators, the directness with which monitoring data inform training decisions and performance evaluations, and the extent to which sport performance depends on technical, tactical, and situational information. Accordingly, between-group differences are best interpreted as differences among monitoring contexts rather than as fixed characteristics of entire sport categories.

The contrast between physical endurance sports and skill-based sports was statistically significant. The standardized DTMS–evaluative anxiety path coefficient was higher in physical endurance sports (β = 0.59) than in skill-based sports (β = 0.31), Δχ^2^(1) = 7.99, *p* = 0.005. This difference may reflect variation in the extent to which training decisions and performance evaluations depend on quantitative monitoring data. Physical endurance sports typically rely heavily on indicators such as heart rate, speed, distance, training load, and recovery status, and these measures are often directly incorporated into assessments of training quality, competitive condition, and load adjustment (Bourdon et al., 2017; Saw et al., 2016). In such contexts, monitoring data may carry substantial diagnostic and evaluative salience. Deviations from expected values may therefore be more readily incorporated into coaching judgments, training modifications, and comparisons among athletes. The relatively direct link between quantitative indicators and subsequent evaluation may help explain why monitoring-related stress was more strongly associated with evaluative anxiety in physical endurance sports.

In skill-based sports, by contrast, performance evaluation typically incorporates a broader range of qualitative and contextual information, including movement quality, technical difficulty, execution consistency, and sport-specific performance demands. Quantitative physiological or workload indicators therefore tend to represent only one component of the evaluative process and may not independently determine judgments of performance quality. Anomalous monitoring results may also be interpreted alongside technical execution and the specific circumstances of training. This greater contextual flexibility may reduce the extent to which individual monitoring indicators are perceived as direct signals of negative evaluation, providing a plausible explanation for the weaker DTMS–evaluative anxiety association observed in skill-based sports.

The pairwise differences involving competitive ball sports were not statistically significant. The DTMS–evaluative anxiety pathway did not differ significantly between physical endurance sports and competitive ball sports or between competitive ball sports and skill-based sports. This pattern may reflect the multidimensional nature of performance evaluation in ball sports. Quantitative indicators such as running distance, speed variation, workload intensity, and recovery status are frequently used, but their interpretation is embedded within tactical roles, positional responsibilities, team coordination, and situational demands. Consequently, monitoring data may carry substantial evaluative value without independently determining overall performance judgments. Although the standardized coefficient for competitive ball sports fell between those observed for physical endurance and skill-based sports, this ordering should be regarded as descriptive rather than as evidence of a stable sport-type gradient.

From the perspective of the Challenge–Hindrance Stressor Framework, the gender and sport-type findings collectively suggest that the psychological significance of monitoring-related demands may depend not only on their intensity but also on their evaluative consequences and the degree of interpretive flexibility available within a given training context (Cavanaugh et al., 2000; LePine et al., 2005). In physical endurance sports, monitoring indicators may function more directly as evaluative signals because of their close integration into assessments of training and competitive condition, whereas skill-based sports may permit greater contextual interpretation of quantitative data. Similarly, the association between monitoring stress and evaluative anxiety may differ across gender groups because comparable feedback can acquire different psychological meanings depending on athletes’ evaluative experiences, self-presentational demands, and perceptions of the consequences associated with performance information. These interpretations remain provisional, however, because the present cross-sectional design cannot establish the mechanisms underlying the observed group differences.

## Conclusions and recommendations

### Conclusion

Drawing on the Technology Stress Generators Model, the Challenge–Hindrance Stressor Framework, and the Job Demands–Resources Model, this study developed and tested a theoretical pathway linking data-driven training monitoring stress (DTMS), evaluative anxiety, psychological exhaustion, and athlete burnout. Based on cross-sectional questionnaire data analyzed using structural equation modeling, bootstrap tests of indirect associations, and multigroup analysis, the following conclusions were drawn:

(1) DTMS was significantly and positively associated with athlete burnout. Athletes reporting higher levels of monitoring-related stress also reported higher levels of burnout. This finding indicates that continuous data collection, metric comparison, and evaluative feedback may represent not only technical components of training management but also psychologically salient contextual demands associated with burnout in data-intensive training environments.
(2) DTMS was significantly and positively associated with evaluative anxiety. When athletes perceive fluctuations in monitoring indicators, rankings, or feedback as carrying implications for coaching judgments, within-team comparisons, or the allocation of valued opportunities, they may experience greater concern about unfavorable evaluation. This finding suggests that the evaluative meaning attached to monitoring information represents an important psychological dimension for understanding the association between DTMS and athletes’ psychological strain.
(3) Evaluative anxiety was significantly and positively associated with psychological exhaustion, and psychological exhaustion was, in turn, positively associated with athlete burnout. Higher levels of evaluative anxiety were accompanied by greater psychological resource depletion, while greater psychological exhaustion was associated with more pronounced burnout symptoms. These findings highlight psychological resource depletion as an important theoretical mechanism for understanding the associations among DTMS, evaluative anxiety, and athlete burnout.
(4) Evaluative anxiety and psychological exhaustion constituted a significant sequential indirect pathway between DTMS and athlete burnout. Higher levels of DTMS were associated with greater evaluative anxiety, which was further associated with greater psychological exhaustion; higher levels of psychological exhaustion were, in turn, associated with greater athlete burnout. Because this study employed a cross-sectional design, this pattern should be interpreted as a theoretically consistent sequential indirect association rather than as evidence of an established temporal or causal chain.
(5) After measurement invariance across gender and sport-type groups had been established, multigroup analyses revealed significant differences in the DTMS–evaluative anxiety pathway by gender and in selected sport-type comparisons. The association was stronger among female athletes than among male athletes and stronger among athletes in physical endurance sports than among those in skill-based sports. Differences involving competitive ball sports and the other two sport categories were not statistically significant. These findings indicate that the strength of the association between DTMS and evaluative anxiety may vary across athlete groups and monitoring contexts. Because the sport categories used in this study represent an operational classification developed for the present data-driven training context, the observed differences should not be interpreted as fixed or universally applicable characteristics of particular sports.

### Recommendations

Based on the observed associations among data-driven training monitoring stress (DTMS), evaluative anxiety, psychological exhaustion, and athlete burnout, training monitoring practices should extend beyond data acquisition and performance evaluation to place greater emphasis on data interpretation, feedback delivery, and athletes’ psychological responses to monitoring. The recommendations below are derived from the statistical associations identified in the present study and should therefore be regarded as practice-oriented implications rather than empirically validated intervention strategies. Their effectiveness requires further evaluation through longitudinal and intervention-based research.

(1) Strengthen contextualized data interpretation and avoid categorical judgments based on isolated indicators. Training data should be interpreted collaboratively by coaches, strength and conditioning specialists, medical staff, and athletes within the context of athletes’ current training conditions, rather than being used to generate immediate categorical judgments such as “good/bad” or “meets/does not meet the standard.” When abnormal heart rate responses, reduced workload, or low recovery scores are observed, interpretation should take into account the training cycle, recent workload, sleep and recovery status, injury-related factors, equipment accuracy, and athletes’ subjective experiences. Athletes should also be given opportunities to explain perceived changes in their condition. Training adjustments can then be considered on the basis of this broader assessment, thereby reducing the likelihood that short-term fluctuations are interpreted as direct evidence of inadequate ability, effort, or training attitude.

(2) Reduce excessive cross-athlete comparisons and prioritize feedback based on individual trajectories. Routine monitoring should place greater emphasis on personal baselines, phase-specific trends, and expected ranges of variation rather than relying predominantly on absolute differences between athletes. Training staff may establish longitudinal monitoring profiles that allow current data to be interpreted relative to athletes’ own historical values and current training phases. Feedback should therefore emphasize descriptive changes, such as “this value differs from your recent baseline” or “your recovery indicators have fluctuated over the past several sessions,” rather than comparative judgments such as “you are performing worse than others” or “you have failed to meet the standard.” The appropriate reference periods and thresholds for individual baselines should nevertheless be determined according to sport-specific demands, training cycles, and relevant professional guidelines.

(3) Interpret anomalous monitoring data cautiously before linking them to training or evaluative consequences. A single anomalous value should first be verified and interpreted in relation to the training phase, physical condition, recovery status, measurement reliability, and the athlete’s subjective experience before decisions are made regarding training modification. When anomalous data coincide with persistent fatigue, impaired recovery, or marked psychological strain, coaches should consider an integrated assessment incorporating training, medical, recovery, and psychological information. The purpose is not to disregard abnormal indicators but to avoid prematurely translating isolated fluctuations into increased training demands, negative performance judgments, or decisions regarding opportunity allocation without sufficient contextual evidence.

(4) Incorporate athletes’ psychological responses into routine monitoring practices. Existing monitoring systems typically emphasize workload, speed, heart rate, and recovery indicators while paying comparatively less attention to athletes’ psychological responses to monitoring itself. Training teams may consider periodically using brief self-report measures or structured communication to identify persistent concerns about monitoring data, evaluative pressure, interpretive burden, psychological fatigue, or difficulties with recovery. When athletes report sustained evaluative anxiety or psychological exhaustion, staff should review how monitoring information is communicated, the extent of public comparison, and the degree to which data are linked to ranking, selection, or opportunity allocation. More explanatory, individualized, and context-sensitive feedback may then be considered. However, the frequency of psychological monitoring and the specific tools employed should be determined according to the training context rather than imposed through fixed schedules or universal thresholds derived from the present study.

(5) Adjust the evaluative weight of monitoring indicators according to sport-specific data-use characteristics. A uniform data-evaluation logic should not be applied across all sport types. In physical endurance sports, quantitative indicators such as heart rate, speed, distance, workload, and recovery status may play a relatively prominent role, but they should still be interpreted in relation to individual baselines, training phases, and sport-specific demands. In skill-based sports, physiological and workload indicators may serve as important supplementary information, while expert judgments concerning movement quality, technical difficulty, and execution consistency should remain central to performance evaluation. In competitive ball sports, running distance, speed, workload, and recovery data should be interpreted alongside tactical responsibilities, playing position, match intensity, team roles, and situational demands. Such differentiated use of monitoring information may help preserve the technical value of data while reducing the likelihood that quantitative indicators are assigned disproportionate evaluative weight.

## Limitations and directions for future research

Several limitations should be acknowledged. First, this study employed a cross-sectional design. The structural pathways and indirect associations identified therefore reflect statistical relationships among variables and cannot establish temporal ordering or causal direction. Future research should examine these relationships using longitudinal designs, cross-lagged panel models, or experimental approaches to provide stronger evidence regarding temporal and causal processes.

Second, all core variables were assessed using athlete self-report questionnaires. Although procedural remedies and statistical assessments were used to reduce and evaluate the risk of common method variance, its influence cannot be completely excluded. Future studies could strengthen methodological triangulation by incorporating multiple data sources, including coach evaluations, behavioral indicators, and objectively recorded monitoring data such as training load, physiological responses, and performance metrics.

Third, several measurement instruments were newly developed or contextually adapted for competitive training environments. Although the present study provided preliminary evidence of satisfactory reliability, convergent validity, and discriminant validity, the stability and generalizability of these measures across independent samples, competitive levels, and sport-specific settings require further examination. In particular, although DTMS and evaluative anxiety, as well as psychological exhaustion and athlete burnout, demonstrated acceptable empirical distinctiveness, these construct pairs remain theoretically adjacent in certain respects. Future research should therefore undertake additional psychometric validation, including replication in independent samples and further examination of construct boundaries.

Fourth, the sample was drawn primarily from competitive training contexts in China, which may limit the generalizability of the findings to other cultural and organizational settings. In addition, the sport categories used in this study represented an operational classification developed specifically to facilitate comparisons across data-driven monitoring contexts. Considerable variation in monitoring practices, data-use intensity, and evaluative criteria may still exist among individual sports within the same category. Future studies should therefore examine whether the observed associations can be replicated across different cultural contexts, training systems, competitive levels, and specific sports.

Finally, because the anonymized dataset did not retain identifiers for specific teams, potential clustering effects among athletes nested within the same training unit could not be estimated. Athletes from the same team may share coaching practices, monitoring procedures, and evaluative climates, which could introduce non-independence into the data. Future research should retain appropriately anonymized team-level identifiers and employ multilevel modeling or other hierarchical analytic approaches to account for nested data structures.

## Data Availability

The original contributions presented in this study are included in this article/Supplementary material, further inquiries can be directed to the corresponding author.
