# Supplementary material for: The association between data-driven training monitoring and athlete burnout: a chain-mediated indirect path between performance anxiety and psychological exhaustion
Source: Front Psychol. 2026 Oct 2;17:1893721. doi: 10.3389/fpsyg.2026.1893721 (PMC13671927; doi:10.3389/fpsyg.2026.1893721)
Supplement: Supplementary file 1 [file Table_2.docx]

**Supplementary Table S1. Items of Study-Specific Measures**

***Working draft note.*** *Section 2.2 of the manuscript reports only representative items rather than the complete administered item set. The items below therefore reconstruct the full scales from the reported construct definitions and examples and should be checked against the original questionnaire before submission.*

| **Construct** | **Item** | **Item wording** |
| --- | --- | --- |
| **Digital Training Monitoring Pressure (DTMP)** | DTMP1 | I feel pressured when my training data are continuously recorded. |
|  | DTMP2 | I feel a psychological burden when my physical condition and training performance are continuously monitored through data. |
|  | DTMP3 | I feel pressured when my training data are frequently compared with expected standards or with those of other athletes. |
|  | DTMP4 | When my training data fall below expectations, I worry that my coach may make a negative judgment about my condition. |
|  | DTMP5 | I feel pressured when training data are used as a basis for evaluating my training performance. |
|  | DTMP6 | When unusual values appear in my training data, I feel that I need to explain them promptly. |
|  | DTMP7 | I need to expend extra effort to explain or respond to unusual changes in my training data. |
|  | DTMP8 | Continually adjusting my training in response to monitoring data creates additional pressure for me. |
| **Evaluative Anxiety (EA)** | EA1 | I worry that poor training data will negatively affect my coach’s evaluation of me. |
|  | EA2 | When abnormal monitoring data appear, I worry that others will think that I am in poor training condition. |
|  | EA3 | I feel nervous when my training data are compared with those of other athletes on the team. |
|  | EA4 | I worry that a decline in my training data will make others question my athletic ability. |
|  | EA5 | When my data are lower than those of other athletes, I worry that this will affect how I am evaluated within the team. |
|  | EA6 | I worry that unfavorable monitoring results may affect my training opportunities or competition selection. |
| **Psychological Exhaustion (PE)** | PE1 | I often feel mentally exhausted when I am continually exposed to training-data feedback. |
|  | PE2 | During training, I need to exert considerable mental effort to maintain concentration. |
|  | PE3 | When my training data are under continuous attention, I find it difficult to relax mentally. |
|  | PE4 | Prolonged attention to training data leaves me feeling mentally drained. |
|  | PE5 | After repeatedly responding to training feedback, I often feel that I have little mental energy left. |
|  | PE6 | After continuously monitoring and interpreting my training data, I find it difficult to recover mentally. |

***Note.*** DTMP = Digital Training Monitoring Pressure; EA = Evaluative Anxiety; PE = Psychological Exhaustion. All items are rated on a 5-point Likert scale ranging from 1 (strongly disagree/not at all true) to 5 (strongly agree/completely true). Higher mean scores indicate higher levels of the corresponding construct.
